# Multi-level variations of lateral habenula in depression: A comprehensive review of current evidence

**DOI:** 10.3389/fpsyt.2022.1043846

**Published:** 2022-10-28

**Authors:** Guang-Ming Zhang, Hong-Yun Wu, Wen-Qiang Cui, Wei Peng

**Affiliations:** ^1^College of Chinese Medicine, Shandong University of Traditional Chinese Medicine, Jinan, China; ^2^First College of Clinical Medicine, Shandong University of Traditional Chinese Medicine, Jinan, China; ^3^Department of Neurology, Affiliated Hospital of Shandong University of Traditional Chinese Medicine, Jinan, China

**Keywords:** lateral habenula, physiopathology, depression, functional projections, synaptic transmission

## Abstract

Despite extensive research in recent decades, knowledge of the pathophysiology of depression in neural circuits remains limited. Recently, the lateral habenula (LHb) has been extensively reported to undergo a series of adaptive changes at multiple levels during the depression state. As a crucial relay in brain networks associated with emotion regulation, LHb receives excitatory or inhibitory projections from upstream brain regions related to stress and cognition and interacts with brain regions involved in emotion regulation. A series of pathological alterations induced by aberrant inputs cause abnormal function of the LHb, resulting in dysregulation of mood and motivation, which present with depressive-like phenotypes in rodents. Herein, we systematically combed advances from rodents, summarized changes in the LHb and related neural circuits in depression, and attempted to analyze the intrinsic logical relationship among these pathological alterations. We expect that this summary will greatly enhance our understanding of the pathological processes of depression. This is advantageous for fostering the understanding and screening of potential antidepressant targets against LHb.

## Introduction

Depression is a neuropsychiatric disorder regarded as the most prevalent crippling and chronic mood disorder (1). The negative symptoms of depression include affective blunting, anhedonia, and social withdrawal, which profoundly affect an individual’s quality of life. The triggers of depression are multifaceted, in addition to the most common stressors, including pain (2), substance use disorders (3), and Parkinson’s disease (PD) (4). These factors complicate the pathophysiology of depression. Treatment of depression concomitant with other chronic diseases is challenging.

**FIGURE 1:**
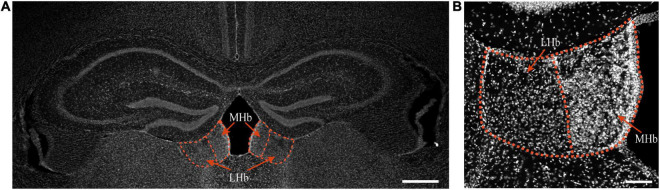
The habenula in mice in physiological state. Top, the location of the MHb and LHb is indicated by red circles and red arrows, bar = 500 μm **(A)**. DAPI staining of the habenula of mice, bar = 100 μm **(B)**.

Although the precise causes and pathophysiology of depression are still unknown, the understandings of the neurobiological mechanisms in depression have rapidly progressed over the last decades (5). Among these, several publications have focused on pathophysiological states and adaptive changes in emotionally regulated brain areas (6). The habenula has received growing interest due to its unique role and essential function in neural mechanisms of depression (7). The habenula is a small bilateral region located in the posterior-dorsal-medial end aspect of the thalamus, which can be classified into two nuclear complexes: the medial habenula (MHb) and the lateral habenula (LHb) (Figure 1). The LHb is a complex and heterogeneous nucleus that receives afferent inputs mainly from the limbic forebrain region and the basal ganglia (8, 9). It sends major glutamatergic projection to the rostromedial tegmental nucleus (RMTg) and inhibits monoaminergic nuclei based on the relay function of RMTg. Furthermore, LHb has attracted considerable interest due to its exceptional location in modulating both the dopaminergic and serotonergic system in the raphe nuclei (10–12) and, in particular, both of which are neurotransmitters that regulate emotions (13, 14). LHb has been widely reported to be strongly associated with depression (8, 15, 16) and has emerged as a crucial determinant of the pathogenesis of depression (17–19).

The dysregulation and major mechanisms of LHb in depression have been well summarized by Browne et al. (20) and Gold et al. (21). However, there are still a lot of aspects remain fragmentary. Few studies have systemically looked at adaptive changes in the LHb at many different levels, especially at synaptic function, and analyzed logical cause and effect relationships between these changes. This review covers the roles of the LHb in depression and the process from physiological activation to pathological hyperactivity. The most recent findings on studies in rodent depression models will be presented from several aspects, including anatomical base, nerve excitability, functional projections, synaptic transmission, and the molecular mechanisms involved. In particular, we attempted to speculate on the complicated pathological process by which risk factors for depression result in a series of pathological alterations in the LHb. This will aid in understanding the detailed mechanisms of LHb in depression and the screening of potential antidepressant targets.

## The anatomical base of depression in lateral habenula

### Asymmetry in lateral habenula in physiological and pathophysiological states

In multiple classes of mammals and humans, the LHb exhibits structural asymmetry, which might be related to differential activation and damage on both sides (22, 23). In contrast to the MHb, the LHb volume on the left side is larger than that on the right side in healthy individuals (24) and patients with major depressive disorder (MDD) (25). Analysis of brain magnetic resonance imaging (MRI) data from patients with MDD has revealed significantly more habenula-thalamic fiber connections on the right side than on the left side (26). This aberrant connectivity has also been observed in subclinical depression, which is considered a harbinger of MDD (27). The posterior parietal thalamus where LHb is located showed increased functional connectivity in resting-state functional MRI of patients with subclinical depression (28). These findings corroborate the essential role of asymmetrical projections to the LHb from thalamic nuclei in depression. Indeed, in addition to its structure, functional asymmetry in the habenula has been reported. Stress activates the LHb asymmetrically (29). The right-side habenula activation in depression is considerably higher than that in healthy individuals; however, the degree of activation on the left side positively correlates with levels of anhedonia (30). In a mouse model of partial transection of the infraorbital nerve (pT-ION), compared to analgesic and anxiolytic effects through suppression of bilateral LHb activity, selective inhibition of glutamatergic neurons in the unilateral (left side) LHb mitigates pT-ION-induced anxiety-like behaviors but fails to alleviate neuropathic pain (31).

It is possible to assume that the asymmetry of the habenula is probably closely linked to the long-term brain stimulation by MDD; however, the exact intrinsic link remains unclear. Perhaps this could be explained by the theory of lateralization of the brain, which organizes brain functions into specific brain hemispheres and is pervasive in vertebrates (32).

### The neural connection network of lateral habenula

#### Inputs

**FIGURE 2:**
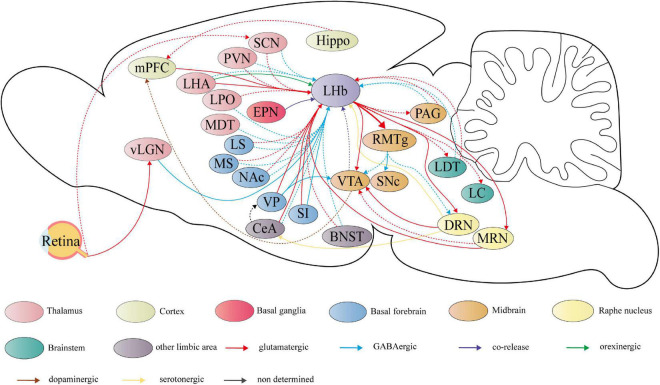
Summary schematic of neural circuits linking the lateral habenula (LHb) to the pathophysiology of depression. The circuits have experimentally proven to regulate depressive-like behaviors, including anhedonia, social disorder, passive coping, and aggressive behaviors are represented by the solid line. Dashed lines represent potential functional circuits, but there is insufficient clear experimental evidence to determine the regulatory role in depressive-like behaviors. LHb receives primarily glutamatergic input (shown in red) and GABAergic inputs (shown in blue). The first source is hypothalamic structures, including the lateral hypothalamic area (LHA), paraventricular nucleus (PVN), suprachiasmatic nucleus (SCN), lateral preoptic area (LPO), and the medial dorsal thalamic nucleus (MDT). The second is the basal forebrain, including the ventral pallidum (VP), nucleus accumbens (NAc), substantia innominate (SI), medial septum (MS), and lateral septum (LS). Other limbic areas, such as central amygdala (CeA), and bed nucleus of the stria terminalis (BNST) are also the sources of LHb input. The medial prefrontal cortex (mPFC) also projects directly to the LHb. Furthermore, LHb receives glutamate/GABA co-releasing projections (shown in purple) from the entopeduncular nucleus (EPN) located in the basal ganglia and GABAergic projections from the thalamic ventral lateral geniculate nucleus–intergeniculate leaflet (vLGN–IGL) located in the visual thalamic region. The vLGN–IGL and the SCN can receive light signaling from retina cells. LHA also sends orexinergic projection (shown in green) to LHb. Based on the strong glutamatergic projections to the rostromedial tegmental nucleus (RMTg), LHb forms a complex network of projections to both dopaminergic nuclei and the raphe nucleus, which the former comprises the ventral tegmental area (VTA) and substantia nigra pars compacta (SNc), and the latter comprises dorsal raphe nucleus (DRN) and median raphe nucleus (MRN). Dopaminergic and serotonergic projections are shown respectively in brown and in yellow. Furthermore, LHb sends glutamatergic projection to the periaqueductal gray (PAG), laterodorsal tegmental nucleus (LDT) and locus coeruleus (LC) and receives feedback projections from these regions. Hippocampus (Hippo), which is strongly related to depression, shows a potential connection with LHb.

The habenula is a conserved and stable bilateral brain structure that is widely present in multiple species (33). It can be further divided into the medial subnucleus (LHbM) and the lateral subnucleus (LHbL), which receives and processes inputs from the limbic brain region and the basal ganglia through the fiber tract of the stria medullaris and serves as the point of intersection of signals from both sources in the hypothalamus (34, 35) (Figure 2). Specifically, limbic inputs from hypothalamic structures such as the lateral hypothalamic area (LHA), paraventricular nucleus (PVN), suprachiasmatic nucleus (SCN) (36), the lateral preoptic area (LPO) (37), and the medial dorsal thalamic nucleus (MDT) (38). The second significant source of neuronal input is the basal forebrain, including, ventral pallidum (VP) (39), nucleus accumbens (NAc) (40), substantia innominate (SI) (41), medial septum (MS) (42, 43), and lateral septum (LS) (44). Central amygdala (CeA) (45) and bed nucleus of the stria terminalis (BNST) (36), which belong to other limbic areas, also project to the LHb. Furthermore, there are projecting neurons from the basal ganglia and cortex, mainly the entopeduncular nucleus (EPN) (46) and medial prefrontal cortex (mPFC) (47). The thalamic ventral lateral geniculate nucleus–intergeniculate leaflet (vLGN–IGL), which is attributed to the visual thalamic region, also projects to the LHb (18). A recent study reveals more LHb-projecting neurons along with the type of transmission through immunohistochemistry and viral tracing methods, which expands our understanding of inputs to the LHb (48).

#### Outputs

On the output side, the downstream projections of the LHb are relatively homogeneous and clear compared to richly sourced inputs (49). Through the fasciculus retroflexus, also known as the habenula–interpeduncular tract, the LHb sends strong projection to GABAergic neurons in RMTg, which is a GABAergic relay for projections to monoaminergic nuclei (50). In addition, the LHb sends glutamatergic projection to the substantia nigra pars compacta (SNc) (51), periaqueductal gray (PAG) (52), locus coeruleus (LC) (53), and laterodorsal tegmental nucleus (LDT) (54) and receives feedback projections from these regions (Figure 2). The LHb also sends reciprocal projections to the ventral tegmental area (VTA) (55), dorsal raphe nucleus (DRN), and median raphe nucleus (MRN) (56–58).

## Lateral habenula hyperactivity during depressive state

### Neural excitation

Lateral habenula neural hyperexcitability can be observed in different rodent models of depression, such as repeated social defeat stress or chronic restraint stress (CRS) (59, 60), early life stress (61), aversive stimuli (62), lipopolysaccharide (LPS) models (59), and learned helplessness (55). Consequently, depressive symptoms can be improved by reducing neuronal activity in the LHb (17) or pharmacological inhibition of LHb function (63). The evoked expression of immediate-early gene-encoded proteins (c-Fos) is one of the markers associated with neuronal activity. Acute stress exposure and chronic social defeat stress (CSDS) resulted in elevated levels of c-Fos expression in mice LHb (64, 65). A more detailed study showed that stress selectively activated one of the LHb subpopulations (66). Approximately 10% of LHb glutamatergic neurons show an opposite inhibitory response to foot shock (67). Compared with mice that exhibited social avoidance from immediately after the repeated social defeat stress, c-Fos expression in the LHb was higher in those that exhibited social avoidance only at later periods (68). This demonstrates that the activity of LHb neurons would perhaps affect different avoidance strategies to stress. The data above show that the stress effects on LHb neurons are more complex than just the activated effect. In human MRI-based studies, patients with depression exhibit right-sided habenula activation, which is associated with more severe depressive symptoms and lack of pleasure (30). These data are in agreement with the asymmetry in the LHb described previously.

Strangely, the use of selective serotonin reuptake inhibitors (SSRIs) led to LHb activation, which contradicts their antidepressant effects (69, 70). One speculation is that the increase in serotonin (5-HT) induced by these SSRIs acts on monoamine receptors with neuronal excitatory effects on the LHb. Sexual dimorphism has been observed in alcohol-induced anxiety and the LHb stress response (65, 71). This may be ascribed to sex differences in the stress-responsive brain regions and related functional networks. The firing rate of VTA-projecting LHb neurons increased specifically in female mice after subchronic variable stress (72). A recent study showed sexual dimorphism of inputs to the LHb in mice, including more excitatory projection neurons in female mice and stronger GABAergic projections to the LHb in male mice (44). In summary, these results indicated a higher sensitivity to stress in females, which matches clinical observations (73). Moreover, the nature of different stressors can also affect sex differences in neural activation patterns of the LHb (74).

It is almost certain that LHb overactivity causes depressive symptoms. Overexcitation of the LHb may result from changes in LHb-projecting neurons, dysregulation of synaptic transmission, and changes in synaptic plasticity.

### Burst firing

Enhanced LHb glutamatergic synaptic transmission and an overall increase in tonic and burst firing are observed in a rat model of early life stress, indicating an increase in intrinsic excitability (75). Except for early life stress, CRS can induce burst firing of action potentials in the LHb neurons, and knocking out p11 reverses this change (60). As a prodromal state and significant inducer of depression, early-stress-induced LHb burst firing is a precursor to depressive symptoms, which could perhaps improve indirect inhibitory input to downstream regions and release neuropeptides (76). The most well-known work is a series of studies from Hu Hailan’s laboratory (19, 77). Hu et al. indicated that *N*-methyl-D-aspartate receptor (NMDAR)-dependent burst firing of LHb neurons was observed in mice with depression phenotypes (77). This process is complicated and requires the participation of low voltage-sensitive T-type Ca^2+^ channels (T-VSCCs) and neuronal resting membrane potentials, of which the latter is regulated by Kir4.1. The LHb burst firing is reversed by ketamine, an NMDAR antagonist (78). A further study by Cui et al. indicated that an increase in depression-like symptoms was accompanied by the upregulation of astroglial Kir4.1 in the LHb (19).

In the study mentioned above, the main outcome measures of the depression-like phenotype were anhedonia. However, in a study by Cerniauskas et al. tonic firing was observed in overactive LHb after chronic stress (79). These mice exhibited different behavioral phenotypes of motivational impairment. Although variations in neuronal potential may have resulted from the different detection methods, implications for LHb tonic firing could not be easily ruled out (61). The difference in the projection subtypes of LHb neurons may lead to varied presentations of depression-like phenotype (79). An important goal of future studies is to define the mechanism of potential changes in the LHb. It is well established that LHb burst firing belongs to a postsynaptic event most probably driven by presynaptic regulation.

## Functional synaptic circuits related to depression symptoms

The important role of the LHb in the pathophysiology of depression is largely due to its rich projection neurons (7). The LHb receives input from stress-responsive and motivation-related upstream regions and emits inhibitory outputs to downstream brain regions associated with monoamine neurotransmitters (20). Stress can activate not only LHb itself but also the nerve projection network for receiving and processing stress, which consists of mPFC, BNST, LHb etc. (80). In fact, neither in a resting state (81) nor under stress (82), LHb plays multiple crucial roles in the network composed of synaptic circuits (Figure 2).

### Excitatory projections to lateral habenula

Lateral habenula neurons are primarily glutamatergic. These glutamatergic neurons receive abundant glutamatergic projections from the limbic forebrain regions and basal ganglia and achieve indirect control of the midbrain monoaminergic nuclei based on strong glutamatergic projections to RMTg (7). Therefore, glutamate is the most basic signaling molecule involved in the neural regulatory functions of the LHb.

#### Basal forebrain

After exposure to stress, different subgroups of parvalbumin (PV)-positive VP neurons generate excitatory output to the LHb and inhibitory output to the VTA, causing behavioral despair/helplessness and social withdrawal, respectively (83). Stress-activated VP neurons targeting different brain regions exhibit different depressive symptoms. Topically applied ketamine and optogenetic inhibition of the VP-LHb circuit rescued the CSDS-induced depression-like phenotype (39). This circuit also contributes to cocaine-withdrawal symptoms (84). The substantia innominate (SI), another subregion of the basal forebrain adjacent to the VP, receives input from the CeA processing aversive emotions and sends glutamatergic and GABAergic projections to the LHb (41, 85). LHb-projecting SI glutamatergic neurons were confirmed to encode nerve activation of the LHb after acute aversive stimuli and mediate depressive-like behaviors after chronic unpredictable mild stress (CUMS) in mice (41). Silencing SI neurons or reward consumption can reduce depressive-like behaviors by inhibiting LHb-projecting SI neurons (41). The NAc can also be activated by stress and showed a significant neuromodulation effect on emotion and depression (65, 86). The interaction between the NAc and the LHb requires further study.

Moreover, glutamatergic projections from LPO neurons can act together with LHb-projecting GABAergic neurons in normal reward and aversion processing (37). Similar situations also exist with projection neurons from MS and to LHb. Co-dominance of LHb neuron by MS GABAergic and glutamatergic axons bidirectionally modulate LHb activity and convey both rewarding and aversive signals to LHb (42, 43). LHb is likely a hub that converting aversive information to negative emotion. Given the dysregulation of the reward system that underlies anhedonia and other depressive symptoms, LHb circuits related to reward aversion warrant intensive study. LS, which is contiguous with the MS, relays stress information to LHb the stress response network through parallel GABAergic and glutamatergic projections (80).

#### Lateral hypothalamic area

The glutamatergic LHA-LHb circuit is an essential node in value processing and escapes behavior after aversive stimuli (87, 88). The LHA transmits stress signals through functional nerve fibers projecting to the LHb, which drives the depression-like phenotype in mice by inducing increased activation and burst firing in the LHb (89). More importantly, a pattern of synaptic potentiation induced by chronic stress was first discovered in the LHA-LHb circuit, and artificial induction of this potentiation can produce depression in naive mice (89).

#### Medial prefrontal cortex

Activation of the mPFC-LHb circuit is involved in passive coping behavior and social dysfunction (90, 91). During negative affective stimuli, increased theta/alpha synchrony within the Hb and prefrontal cortex-habenula network has been observed in human participants (92). The lack of extracellular ATP activates LHb-projecting mPFC neurons through disinhibition of mPFC GABAergic interneurons, resulting in depressive-like behaviors in a mouse model of depression induced by CSDS or *IP3R2*-null mutation (47). P2 × 2 in mPFC GABAergic interneurons is a mediating molecule involved in this process.

#### Median raphe nucleus

Glutamatergic LHb-projecting MRN neurons can be activated by aversive stimuli and promote burst firing in the LHb (56). This activation is aversive and drives aggressive behavior and anhedonia. Furthermore, MRN connect with the LHb, VTA, PFC, and hippocampus (Figure 2), making it a key point for the negative experience and related long-term memory formation (56).

#### Paraventricular nucleus

Glutamatergic vasopressin-expressing magnocellular neurons in PVN project directly to the GABAergic interneurons in LHb (93). This circuit can be activated physiologically by water deprivation and inhibit the LHb functional output, resulting in decreased freezing and immobility during innate fear and behavioral despair assessments (93). These findings imply the regulatory potential of vasopressin-expressing PVN neurons in abnormal outputs of LHb during depressive state.

#### Suprachiasmatic nucleus

As the principal circadian pacemaker in mammals receiving light information from the retina, the SCN can innervate the LHb and largely determines the circadian oscillations of neuronal activity and firing pattern in the LHb (94, 95). Moreover, the SCN is likely to modulate the clock gene in LHb, mainly *Per1* and *Per2*, which have been demonstrated to be directly correlated with the day-night variation of depressive-like behaviors in rodent depression models (96, 97). An in-depth study of the SCN-LHb circuit would be important for elucidating the underlying mechanisms of the day-night symptom fluctuation and light therapy in patients with depression or bipolar disorder.

#### Other glutamatergic projections

Another glutamatergic projection worth studying is the BNST-LHb. BNST is considered as a way station between stress regions and brain reward centers (98). Despite the small number of relevant studies, it cannot be denied that BNST is the connecting point receiving stress signals and conveying information to LHb, which might promote stress-induced anxiety (99). Similar effects can be found in the LHb-LDT circuit. Activation of LHb glutamatergic input to LDT GABAergic interneurons can generate fear-like responses, and prolonged activation of interneurons in LDT can induce anxiety-like behaviors (54). LDT is also bidirectionally connected with the LHb and plays a potential role in processing aversive information (100).

The function of reciprocal excitatory projections between the PAG and LHb has not been fully elucidated (44, 52). In a study of patients with irritable bowel syndrome with chronic recurrent abdominal pain and negative emotions as the major symptoms, abnormal enhancement of the resting-state functional connectivity of the LHb-PAG indicated pain-induced negative emotions are possibly related to the LHb-PAG circuit (101). LHb also sends glutamatergic projection to the LC, which is the primary source of norepinephrine in the central nervous system (CNS) and plays a critical role in pain-related anxiety or depression (102). Intensive studies on these two regions contribute to the understanding of how LHb is involved in processing the emotional component of chronic pain.

### Inhibitory projections to lateral habenula

#### Glutamate/GABA co-releasing neurons

Extrinsic GABAergic inputs are the major inhibition mechanisms in the LHb. Most of the current evidence indicates that one of the main contributors of GABA in the LHb is glutamate-GABA co-releasing projecting neurons from the EPN and VTA (46, 103, 104). The distribution of vesicular glutamate transporter 2 (VGluT2), vesicular GABA transporter (VGAT), and glutamic acid decarboxylase (GAD) were determined in these neurons by *in situ* hybridization. GABA and glutamate are released presynaptically into the respective synaptic vesicles (103). In particular, the balance of GABA/glutamate signaling was skewed toward a reduction of GABA in EPN-projecting co-releasing neurons of rats with congenital learned helplessness, which can be reversed by SSRI treatment (46). These phenomena are likely one of the reasons for the increased activity of the LHb among learned helplessness (55). Chronic exposure to stress can induce a depressive-like phenotype of motivational impairments through synaptic adaptations in excitatory EPN inputs to VTA-projecting LHb neurons, including increased presynaptic release probability and upregulation of GluR2-lacking α-amino-3-hydroxy-5-methyl-4-isoxazole-propionic acid receptor (AMPAR) (79). This mode of GABA/glutamate balance regulation has also been observed in cocaine-withdrawal symptoms. The decrease in VGAT in LHb-projecting EPN neurons switches GABA/glutamate signaling from balance to excitation, and cocaine-withdrawal symptoms are reversed by VGAT overexpression (105).

A recent study showed that glutamate/GABA co-packaging in LHb-projecting EPN neurons is regulated by 5-HT or adenosine receptor agonists (104). Moreover, hyperactive LHb may decrease the co-release of VTA-projecting inputs via the inhibition of glutamatergic projections to the VTA (106). Given the abundance of reciprocal projecting neurons between the LHb and VTA, mutual regulation requires further study (107).

#### Other GABAergic projections

Additionally, the medial portion of the LHb receives GABAergic inhibitory inputs from the basal forebrain (40, 108), LPO (37), VP (109), and PV-positive neurons within MDT (38). Overall, inhibitory innervation in the LHb encodes a reward (110). vLGN–IGL, a visual thalamic region, provides a GABAergic input to LHb. This inhibitory pathway receives light signaling via retinal ganglion cells expressing M4-type melanopsin and represses LHb activity, which is one of the mechanisms underlying the antidepressant effect of light therapy (18). Indeed, the vast majority of glutamatergic projections to the LHb are accompanied by parallel GABAergic axons (44) (Figure 2). Like co-releasing neurons, the imbalance between glutamatergic projections and GABAergic projections is probably the pathogenesis of depression and other mood disorders.

### Other regulatory projections

#### Dopaminergic projections

Reciprocal feedback projections between LHb and dopaminergic nuclei, mainly the VTA and SNc, are the main connections between LHb and the dopamine system (111, 112).

The activity of VTA dopaminergic neurons is inhibited when the LHb is activated by stress or electrical stimulation and is involved in the regulation of reward-related behavior (113). This inhibitory effect is achieved through indirect projections via GABAergic RMTg neurons (114). Selective inhibition of VTA neurons has been confirmed to rapidly produce depression-like behaviors, including behavioral despair and anhedonia (115). This suggests that the LHb-RMTg-VTA neural circuits may lead to depression, although this neural circuit appears unaffected by chronic mild stress (116). Furthermore, direct glutamatergic projection from the LHb to the VTA has been observed (117). This excitatory projection does not contradict the indirect inhibition of VTA dopamine neurons by the LHb. A higher release probability at synapses on VTA-projecting LHb neurons enhanced learned helplessness behaviors in the acute or congenital learned helplessness rat model of depression (55). One study indicated that the LHb-VTA circuit favors increased immobility in the tail suspension test rather than anxiety or anhedonia (79). Individual differences in behavior and neurophysiological factors may result in different results (79). Furthermore, LHb neurons can activate VTA dopamine axons projecting to the mPFC (16). Similar to RMTg, VTA is also a relay of LHb to control the neurons of other regions, including upstream regions of LHb.

An additional associated dopaminergic neural circuit is the LHb-RMTg-SNc. RMTg shifts excitatory glutamatergic projection of the LHb into inhibitory GABAergic projection to dopamine neurons in SNc, which has been confirmed to be involved in aversive and despair-like behaviors (51). Inhibition of this pathway reversed behavioral despair in CRS depression model mice (51). In a series of studies by Liu et al. lesions of the SNc in rats could generate a series of consequences in the LHb, including an increased firing rate and hyperactivity of LHb neurons and anxiety-like and depressive-like behaviors (106, 118, 119).

#### Serotonergic dorsal raphe nucleus projections

The reciprocal connectivity between the LHb and DRN is the main 5-HT regulatory pathway in the LHb (120). More specifically, on the one hand, there is a glutamatergic projection from LHb to the GABAergic interneurons and 5-HT neurons in the DRN (121). The RMTg is also a major GABAergic relay between the LHb and the DRN (122). According to brain-wide imaging data in zebrafish exposed to a stress source, passive coping strategies presented progressive activation of neurons in the ventral (lateral) habenula in terms of neuronal dynamics, and downstream neurons in the 5-HT raphe nucleus are suppressed by these activations (123). The discrepancy in the rhythmic discharges of LHb cells projecting to the DRN between stress-susceptible mice and stress-resilient mice led to different reactions of the two types of mice to CSDS (124). DNA hypomethylation in the LHb has been shown to regulate emotional states by inhibiting DRN 5-HT projecting neurons (125). Correspondingly, improved depressive-like behavior in rats and higher 5-HT levels in the DRN after intra-LHb microinjection of a substance P receptor antagonist showed antidepressant potentials in this circuit (126). A recent study showed that one subpopulation of LHb neurons project directly to glutamatergic DRN neurons projecting to VTA dopamine neurons and mediate social instigation-induced aggressive behavior in male mice (127).

In contrast, 5-HT neurons in the DRN project to the LHb and presynaptically inhibit LHb excitatory neurons, which depressive-like behaviors in the CUMS rat model were improved by optogenetic activation of DRN-LHb projections (58). One study suggested the existence of a 5-HT projection from DRN to GABAergic interneurons of the CeA, with glutamatergic neurons in the latter projecting directly to the LHb (128). Inhibition and activation of the pathway separately produced and decreased pain-related depression-like behaviors in a mouse model of chronic pain (128). Increased LHb-DRN pathway activity may serve as one of the underlying mechanisms of the comorbidity of pain and depression (13).

#### Orexinergic lateral hypothalamic area projections

As the sole source of orexin in CNS, LHA orexin neurons project to the LHb, releasing orexin into GAD2-expressing LHb neurons and driving aggressive behaviors in male mice (129, 130). A further investigation showed that LHA orexin neurons project not only to GAD2-expressing neurons but also to glutamatergic neurons directly (131). The LHA*^Orx^*-LHb*^Glu^* circuit can be activated by social stress, and pharmacogenetic activation of orexinergic signaling or optogenetic activation of this circuit can rescue depressive-like and anxiety-like behaviors induced by CSDS (131). The LH*^Orx^*-LHb^*GAD*2^ and LHA*^Orx^*-LHb*^Glu^* circuits act as regulators of different dimensions of social behavior, although the potential connections and interactions between the two remain unknown.

## Changes in lateral habenula synaptic transmission during the depression state

Synaptic transmission is the vital physiological basis of the LHb mechanism in depression (110). Synaptic transmission profoundly affects the intrinsic excitability and potential levels of LHb neurons. Dynamic changes in synaptic regulate the balance of excitation and inhibition, including tentatively potentiated or attenuated synaptic efficacy and long-term improved postsynaptic receptor sensitivity or long-term greater presynaptic release. Since depression is a chronic pathological process, long-term presynaptic or postsynaptic plasticity is more dominant, namely long-term potation, including long-term potentiation (LTP) and long-term depression (LTD). Although the majority are glutamatergic, LHb neurons regulate a variety of neurotransmitters through abundant projection neurons (111). Following sections discuss many receptors in the LHb with modulatory effects on mood and depression, which is summarized in Table 1.

**TABLE 1:** Neurotransmitter receptors in LHb that can regulate depression-like behaviors.

Receptor type	Location	Animal	Drug	Receptor function	Model	Behavior	References
NMDAR	Postsynaptic	C57BL/6 mice	Ketamine	↓	Congenitally learned helpless/ chronic restraint stress	Reversed depression-like behaviors in the FST and SPT induced by burst firing	(77)
GluN2B-containing NMDAR	Postsynaptic	Swiss albino mice	Ro25-6981	↓	Ethanol withdrawal	Alleviated anxiety-like behavior in EPM in ethanol withdrawn mice	(139)
GluN2B-containing NMDAR	Postsynaptic	C57BL/6J mice	Rislenemdaz	↓	Chronic restraint stress	Improved despair-like behavior in TST and depression-like behaviors in the FST and SPT	(140)
CP-AMPARs	Postsynaptic	SD rats	Naspm	↓	6-OHDA lesions of the SNc	Improved depression-like behaviors in the FST and SPT	(144)
AMPARs	Postsynaptic	SD rats	(S)-AMPA	↑	6-OHDA lesions of the SNc	Improved depression-like behaviors in the FST and SPT	(143)
AMPARs	Postsynaptic	SD rats	NBQX	↓	6-OHDA lesions of the SNc	Promoted depression-like behaviors in the FST and SPT	
GABA_*A*_Rs	Postsynaptic	SD rats	Muscimol hydrobromide	↑	6-OHDA lesions of the SNc	Improved depression-like behaviors in the FST and SPT	(151)
			Picrotoxin	↓		Promoted depression-like behaviors in the FST and SPT	
GABA_*B*_Rs	Postsynaptic	C57Bl/6J mice	LB-100 (inhibitor of PP2A)	↑	Foot-shock exposure	Improved depression-like behaviors in the FST and SPT	(153)
					Learned-helplessness	Improved depression-like behaviors in the SPT and increased the rate of failure to escape after foot shocks	
GABA_*B*_Rs	*	C57BL/6 mice	Baclofen	↑	Chronic social defeat stress	Relieved the social avoidance symptoms and the decreased sociability in the stress susceptible group	(64)
			CGP36216	↓			
D1-like dopamine receptor	Presynaptic	SD rats	SKF 38393	↑	Normal rats	Increased anxiety-like behavior in OFT and decrease the depression-like behaviors in FST	(156)
			SCH 23390	↓			
5-HT 4 receptors	*	SD rats	BIMU-8	↑	6-OHDA lesions of the SNc	Improved depression-like behaviors in the FST	(163)
			GR113808	↓			
5-HT 7 receptors	*	SD rats	AS19	↑	6-OHDA lesions of the SNc	Improved depression-like behaviors in the FST and SPT	(164)
			SB269970	↓		Improved depression-like behaviors in the FST and SPT	
5-HT 2C receptors	*	SD rats	Ro60-0175	↑	6-OHDA lesions of the SNc	Promoted depression-like behaviors in the FST and SPT	(165)

The upregulation and downregulation of receptor functions are indicated by ↑ and ↓ arrows, respectively. An asterisk indicates that the distribution of receptors still ambiguous. FST, forced swim test; SPT, sucrose preference test; EPM, elevated plus maze; TST, tail suspension test; OFT, open field test.

### Glutamatergic system

#### Abnormal changes in glutamatergic synaptic transmission

Glutamate is a major neurotransmitter in the CNS. Most neurons in the LHb are glutamatergic (132). Recently, many studies have focused on aberrant synaptic transmission in depression (133, 134). Stress is one of the most severe risk factors in the pathophysiology of MDD and can profoundly regulate glutamatergic synaptic transmission in the LHb. After exposure to CUMS, the results of metabolite analysis showed that glutamate levels were dramatically increased in the LHb of rat (135). Several studies have shown that glutamate transport and clearance barriers can induce depression-like behaviors in rats (118, 136). Early life stress can also enhance glutamatergic synaptic strength through postsynaptic and presynaptic mechanisms, changing the excitation/inhibition (E/I) balance of LHb neurons and persisting into adulthood (75).

Notably, an individual study has also shown a decrease in glutamatergic synaptic transmission after stress (137). This downregulation led to not depressed mood but cognitive deficits accompanied by a postsynaptic decrease in GluA1 AMPAR. Given that cognitive impairment is a frequent complaint in patients with depression (138), two opposing stress-induced glutamatergic synaptic transmissions are likely to be present in the LHb simultaneously.

#### The functional regulation of glutamatergic receptors

The vast majority of the regulatory strength of glutamatergic synapses in the LHb is dependent on NMDAR and AMPAR. Foot-shock-induced excitation of glutamatergic neurons in the LHb required the involvement of NMDAR and AMPAR (67). Local injection of GluN2B-containing NMDAR antagonists or the specific GluN2B antagonist rislenemdaz into the LHb has been demonstrated to improve alcohol-induced anxiety-like behaviors (139) and CRS-induced despair-like behaviors (140). In addition to the aforementioned short-term regulation, NMDAR can also produce LTD in the LHb. The activation of synaptic and extrasynaptic NMDARs containing GluN2A or GluN2B leads to LTD triggered by low frequency stimulation in the LHb (141). Therefore, synaptic NMDA receptor-dependent LTD could be blocked by CRS and may be one of the complex pathological processes of stress-related depressive symptoms (141).

AMPAR is classified into calcium-permeable GluA2-lacking (CP-AMPAR) and calcium-impermeable GluA2-containing (CI-AMPAR) (142). Activation of neurons expressing CI-AMPAR or blockade of neurons expressing CP-AMPAR contributes to an increase in the level of extracellular DA and 5-HT in the mPFC, leading to the improvement of the depression-behaviors of a PD rat model induced by lesions of SNc (143, 144). The two types of neuronal may vary widely in functional features, which may be attributed to differences in targeted neurons (143). Withdrawal-induced symptoms, such as depression, could be regulated by LHb AMPAR (145). Similar to NMDAR, LHb AMPAR can participate in the long-term regulation of excitatory synapse. CP-AMPARs are necessary to activate NMDAR-dependent LTD (141). Although enhancement of LHb AMPAR signaling can increase bursting activity during emotional processes of depression, local injection of the AMPAR blocker into the LHb did not produce a rapid antidepressant effect, similar to the NMDAR blocker (77).

### GABAergic system

The internal inhibition of the LHb neurons has always been worth discussing. One study suggested that GAD2-expressing neurons in the LHb could suppress LHb neuronal activity through GABA release to control aggressive behaviors in male mice (129), although these neurons have been shown to release glutamate rather than GABA, targeting the midbrain (146). The lack of VGAT in LHb neurons renders the release of GABA unclear (147). In summary, GAD2-expressing neurons in the LHb probably release more than one neurotransmitter (148). The finding of locally targeting inhibitory LHb neurons that express PV also further identified internal inhibition in the LHb (38). The density of PV neurons decreased in the LHb of adult mice after early life stress (149). PV-positive neurons are present not only locally but also in projecting neurons (mainly inhibitory) (83, 150). The blockade of GABA transporter 1 (GAT1) or GABA transporter 3 (GAT3) in the LHb upregulated extracellular GABA levels and caused greater GABAergic inhibition, downregulating LHb neuronal activity and firing rate of LHb neurons, resulting in an improvement in depression-like behaviors in PD rats (119).

Stress inhibition in a small portion of LHb neurons requires GABA_*A*_Rs (67). Intra-LHb infusion of muscimol (an agonist of GABA_*A*_R) decreased the firing rate of LHb neurons and produced antidepressant-like effects in rats with PD (151). Activation of GABA_*A*_Rs can decrease neuronal firing in the LHb (152). During the resting state, GABA_*B*_Rs are involved in the physiological suppression of LHb activity. This inhibition was attenuated by stress-induced blunting of GABAB-GIRK signaling, in which protein phosphatase 2A (PP2A) inhibition reversed the changes in GABAB-GIRK signaling and decreased depression-like phenotype (153). Innate differences in GABA_*B*_Rs expression levels determine sensitivity to social stressors in mice (64). A recent study showed that GABA-LTD is regulated by presynaptic GABA_*B*_Rs in mice and can be disturbed by social isolation (154). Notably, the injection of agonists and antagonists of GABA_*B*_Rs resulted in the remission of stress-induced social withdrawal symptoms (64). This contradictory result may be due to the functional divergence of presynaptic and postsynaptic GABA_*B*_Rs.

### Dopamine system

Changes in the concentrations of dopamine and 5-HT were observed across parts of the mesocorticolimbic region in rats with neonatal habenula lesions (155). Spontaneous activity in the LHb is enhanced by dopamine receptor activation (156). Disturbance of dopamine subtype 1 receptor (D1R) function contributes to increased anxiety-like behaviors and decreases depressive-like behaviors (157). The use of dopamine D_4_ receptor agonists or antagonists caused alterations in depression-like behavioral responses, and different doses showed opposite results (158). The analyses of dopamine neurotransmission-related proteins in patients with MDD also showed the implication of D1Rs in mediating antidepressant responses (159).

### Serotonergic system

There are seven groups of 5-HT receptors (1–7), and approximately 15 subtypes are involved in the regulation of neuronal activity and multiple behaviors (160). Peripheral administration of the respective agonists activates 5-HT 2A receptors located mainly in astrocytes and 5-HT 2C receptors located in neuronal cells, which induced mixed excitatory/inhibitory effects in LHb neurons (161). Importantly, blocking 5-HT 2A receptors within the LHb has been shown to inhibit hyperexcitable LHb in depressive states (161). Activation of presynaptic 5-HT 1B receptors decreased excitatory inputs to the LHb (162). This was likely due to their differential distribution. The activation of 5-HT receptors, including 2C, 4, and 7 subtypes, was shown to induce increased discharge activity in LHb neurons, lower monoamine levels in related brain regions, and increase depressive-like behaviors in a PD rat model (163–165). According to the functions of presynaptic 5-HT 1B receptors, apart from other subtypes, 5-HT receptors postsynaptically or presynaptically localized may have different functions.

### Opioid system

The habenula complex is the predominant site of expression of the mu-opioid receptor (MOR) (132, 166, 167). After selective excitation of MOR, whole-cell patch-clamp recordings in rats showed inhibition of LHb neurons via postsynaptic hyperpolarization and presynaptic inhibition of glutamate release (168). From a functional perspective, the latest study indicated that nociceptive stimuli activated the LPO projection to the LHb, producing hyperalgesia and related aversive emotions, which negative emotions can be strongly inhibited by specific activation of the LHb MOR, leading to the mitigation of the above symptoms (169). This showed that the LHb MOR function improves negative emotional experiences in persistent neuropathic pain.

The kappa-opioid receptor (KOR) was also detected in the LHb (170). Functional KOR signaling in the LHb was first discovered by Simmons et al. There is bidirectional modulation of KOR in LHb neuronal excitability in two types of neuron subpopulations by altering presynaptic glutamate release and presynaptic GABA release (171). This modulation relies on intact fast synaptic transmission and is inhibited by decreased KOR expression induced by early life stress (171). The LHb is a critical brain region for negative emotions during opioid abstinence (172). As one of the antireward-aversion centers, the habenula drives negative emotions induced by opioid withdrawal. Opioid receptor activation supports the antidepressant effects of ketamine (173).

## Molecular mechanisms of lateral habenula activity regulation and synaptic function in depression

### Depression-related neuropeptides in lateral habenula

#### Neuropeptide Y

Neuropeptide Y (NPY) is a peptide neuromodulator encompassing five histologic subtypes. It is the most prevalent and extensively distributed neuropeptide in the mammalian brain and plays an essential role in depression and stress (174, 175). Activation of neuropeptide Y1 receptors leads to inhibition of excitatory neurotransmission in the vast majority of LHb neurons via the phospholipase C/protein kinase C-dependent pathway, while potentiation occurs in a small population of neurons (176, 177). Despite this overall inhibitory activity, whether NPY in the LHb can suppress hyperactive LHb to improve depressive symptoms remains to be investigated. In fact, NPY expression has been detected in widely upstream regions of the LHb, although the current results do not provide evidence that NPY modulates synaptic strength and upstream projections (178).

#### Galanin

Multi-species anatomical studies have shown the distribution of ganglion-like immunoreactive fibers in the habenula (179, 180). Galanin (1–15) can improve the antidepressant-like effects of fluoxetine in an olfactory bulbectomy model of depression in rats, accompanied by activation of a functional network that includes the LHb, VTA, and DRN (181).

#### Pituitary adenylate cyclase-activating polypeptide

The neuropeptide pituitary adenylate cyclase-activating polypeptide (PACAP) is expressed in a small population of neurons located in the LHb (182). Chemically selective activation of PACAP-expressing neurons in the LHb can reduce fear and anxiety-associated behaviors (183). This is different from the activation effect of most LHb neurons, which is likely related to internal inhibition of the LHb. PACAP may be expressed in GAD2-expressing LHb neurons, which have been confirmed to inhibit the activity of most LHb neurons (129, 184). Given the above, PACAP might be a good breakthrough point in determining internal inhibition in the LHb.

### Depression-related molecular in lateral habenula

#### Ca^2+^/calmodulin-dependent protein kinase II

Ca^2+^/calmodulin-dependent protein kinase II (CaMKII) regulates synaptic transmission mainly by postsynaptic mechanisms that can be activated by physical stress (185). CaMKII promotes the phosphorylation of S831 in the GluR1 subunit (186, 187) and recruits AMPAR at synapses by inhibiting the diffusion of surface AMPARs at synaptic sites (188). Enhanced AMPAR upregulates glutamatergic synaptic transmission and LHb activity. Numerous studies have shown that upregulation of LHb βCaMKII could cause depressive-like symptoms in rodents, including learned helpless rats (17) and thyroidectomy rats (189), and alcohol-withdrawal rats (187). Inhibition of αCaMKII neurons in the LHb improves depressive symptoms (18). In summary, as the hallmark of LHb activation, CaMKII and LHb activities are tightly linked.

High-frequency stimulation, similar to burst firing, might open the Ca^2+^-permeable NMDA receptor channel, leading to the elevation of postsynaptic Ca^2+^/CaM complex concentration and enhancement of CaMKII function (186). More importantly, due to the persistence of kinase function activity, CaMKII participation might transform transient action potentials or burst firing into LTP, potentially an underlying mechanism of long-standing depressive symptoms in MDD (190).

#### Endocannabinoids

As an important signaling molecule in emotion regulation and synaptic transmission, endocannabinoids (eCB) decrease presynaptic glutamate release probability in the LHb (191). After chronic and intermittent cannabinoid receptor agonist administration, the mouse habenula showed a decrease in cerebral metabolism and weakened functional connectivity with upstream regions, mainly the PFC and basal ganglia (192). This weakened the strength of excitatory projections from these regions to the LHb. In addition to the decrease in excitatory input, another mechanism of eCB-dependent inhibition of activity is an increase in the probability of presynaptic GABA release, which depends on eCB-CB1 receptor signaling (193). More specifically, the expression of CB1Rs in VTA GABAergic neurons allows eCB signaling to regulate inhibitory inputs to the LHb (194). The LHb synapses are repressively regulated in the normal state and are presented as LTD. In summary, similar to GABA_*B*_Rs, LHb eCB signaling maintains LTD via regulatory disynaptic projections during physiological states. LHb eCB signaling is expressed in astrocytes in addition to neurons and has been identified as a new therapeutic strategy for MDD (195).

A previous study indicated that low- and moderate-frequency stimulations maintain eCB-dependent LTD, which likely corresponding to silent or tonic firing during the LHb resting state (191). Exposure to an extreme stressor disturbs eCB-dependent LTD, which could be explained by the fact that LHb neurons produce burst firing after activation, predominantly by aversive or stressful stimuli. Synaptic transmission subsequently transforms from eCB-dependent and NMDAR-dependent LTD to CaMKII-dependent LTP. Recovery of eCB-dependent LTD after αCaMKII inhibition supports this hypothesis (191). These conjectures could associate the electrophysiological properties of the LHb and synaptic plasticity and could be interpreted as one of the possible roles of LHb burst firing. However, as logical as these conjectures, insufficient support for this possibility is currently lacking.

## Summary and future perspectives

**FIGURE 3:**
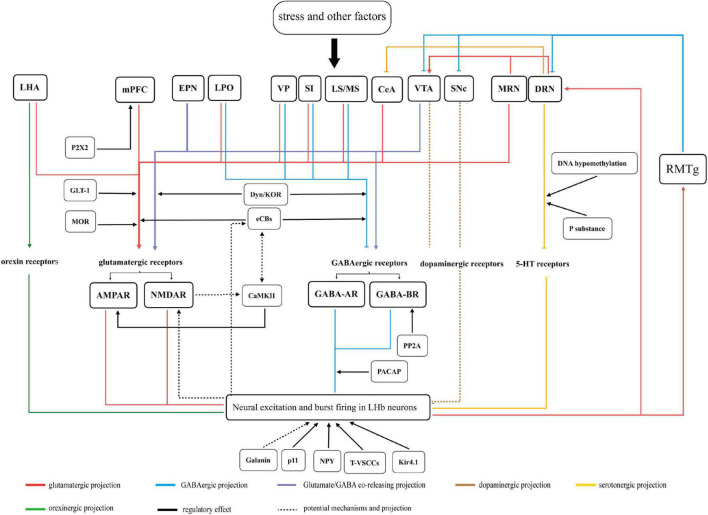
A summary figure of the LHb variations and related mechanisms in depression is discussed in this review. Stress and other factors act on upstream regions. The anomalous projections from the upstream regions to LHb acts on various neurotransmitter receptors in LHb neurons. This results in neural excitation and burst firing in LHb neurons. Subsequently, overactive projecting neurons in LHb mediate dysfunction in monoaminergic nuclei via direct or indirect projections. Moreover, these downstream regions also project to LHb or its upstream regions, which are connected to form a network of neural projections. All the above factors eventually culminate in depressive-like symptoms in rodents. Related molecules and neuropeptides act on input neurons, receptors, and neurons in LHb and play different regulatory roles in the pathological processes of depression. The functional significance of parts of the regions remain uncertain are not represented in figure.

This review provides a comprehensive view of the role of LHb in the mechanisms of depression (Figure 3). Stress likely preferentially activates upstream regions and produces anomalous projections to the LHb, leading to presynaptic and postsynaptic events through the action of various neurotransmitter receptors. This results in postsynaptic action potentials, mainly burst firing in the LHb neurons, in turn, driving sustained neuronal activation, which induces physiological changes at the molecular level. Subsequently, overactive projecting neurons in the LHb mediate dysfunctional inhibition of monoaminergic nuclei. These changes contribute to the dysfunctional homeostasis of monoamine levels in the brain. More importantly, synaptic plasticity and adaptive modulation of receptors and related molecules transform temporary firing into persistent overactivity, which produces prolonged symptoms of depression. In addition, most secondary depressive symptoms caused by chronic pain, substance use disorders, and PD can be attributed to aversive activation or depletion of monoamines in the LHb, which increases the difficulty of treating depression. Some specific regions, including the PAG, LC, CeA, and DRN, perhaps receive pain signals and transmit signals to LHb, resulting in pain-related negative emotion. The dysregulation of projections between LHb and the VTA/SNc, particularly subsequent dopaminergic-system dysregulation, might be the crucial point of PD-induced depression symptoms.

Given LHb has been suggested as a key node in the pathological processes of depression, a growing number of researches are devoted to exploring new treatment strategies acting on LHb. Deep brain stimulation (DBS) of the LHb has received increasing attention since it showed excellent efficacy on patients with treatment-resistant depression (196). LHb DBS has been confirmed that can improve depressive-like behaviors through heightened levels of monoamines (197), enhanced synaptic efficacy (198), and the regulation of depression-related molecular (199). Notably, similar to the complex effect of stress on LHb, DBS potentially alters the functional connectivity networks centered on LHb (200). This presented paper contributes to expanding our understanding of LHb DBS in more aspects. Meanwhile, studies on LHb DBS provide a clear basis for better elucidating the pathogenesis of LHb in depression.

**FIGURE 4:**
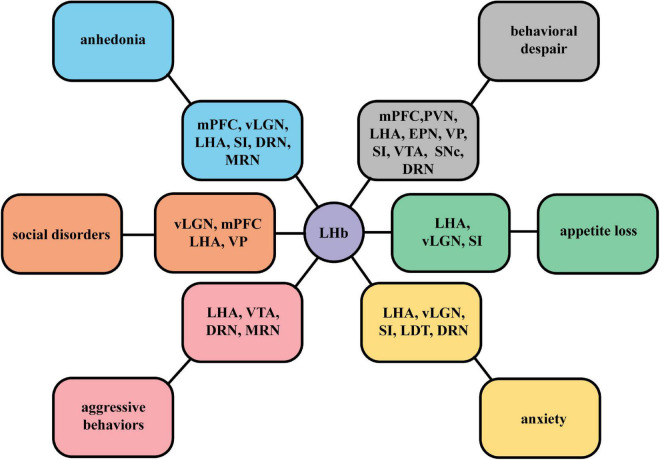
A summary figure of specific mood symptoms dominated by the LHb in different neural circuits. According to specific behavioral manifestations in studies on LHb circuits, we summarize different mainly depressive symptoms mediated by the neural projections between the LHb and different nuclei, including anhedonia, social disorders, behavioral despair, aggressive behaviors, appetite loss, and anxiety.

The neural connections between the LHb and different nuclei mediate different mood symptoms. According to various behavioral methods in studies mentioned in section “Functional synaptic circuits related to depression symptoms,” we summarize mainly depression-like behaviors dominated by the LHb in different neural circuits (Figure 4). These input and output neurons are likely to be targeted to a particular subset of the LHb. It should be noted that we observed the complexity of functional cell subpopulations in LHb neurons. Specific molecular markers, such as parvalbumin, enable their identification. The functional identification of a subpopulation of these neurons is required. Through a systematic review, we expect to contribute to the accurate delineation of pathological processes in the LHb and the screening of therapeutic targets for depression.

## Author contributions

G-MZ wrote the manuscript. WP, W-QC, and H-YW reviewed the literature. WP and W-QC modified the language. G-MZ and H-YW made contributions to the drawing of the figures and table and the revision of the manuscript. All authors contributed to the article and approved the submitted version.

## References

[1] Dean J Keshavan M. The neurobiology of depression: an integrated view. *Asian J Psychiatry.* (2017) 27:101–11. 10.1016/j.ajp.2017.01.025 28558878

[2] Maallo AMS Moulton EA Sieberg CB Giddon DB Borsook D Holmes SA. A lateralized model of the pain-depression dyad. *Neurosci Biobehav Rev.* (2021) 127:876–83. 10.1016/j.neubiorev.2021.06.003 34090918 PMC8289740

[3] Calarco CA Lobo MK. Depression and substance use disorders: clinical comorbidity and shared neurobiology. *Int Rev Neurobiol.* (2021) 157:245–309. 10.1016/bs.irn.2020.09.004 33648671

[4] Chuquilín-Arista F Álvarez-Avellón T Menéndez-González M. Prevalence of depression and anxiety in Parkinson disease and impact on quality of life: a community-based study in Spain. *J Geriatr Psych Neur.* (2020) 33:207–13. 10.1177/0891988719874130 31597514

[5] Kaltenboeck A Harmer C. The neuroscience of depressive disorders: a brief review of the past and some considerations about the future. *Brain Neurosci Adv.* (2018) 2:1864960677. 10.1177/2398212818799269 32166149 PMC7058215

[6] Post RJ Warden MR. Depression: the search for separable behaviors and circuits. *Curr Opin Neurobiol.* (2018) 49:192–200. 10.1016/j.conb.2018.02.018 29529482 PMC6042519

[7] Hu H Cui Y Yang Y. Circuits and functions of the lateral habenula in health and in disease. *Nat Rev Neurosci.* (2020) 21:277–95. 10.1038/s41583-020-0292-4 32269316

[8] Proulx CD Hikosaka O Malinow R. Reward processing by the lateral habenula in normal and depressive behaviors. *Nat Neurosci.* (2014) 17:1146–52. 10.1038/nn.3779 25157511 PMC4305435

[9] Yang Y Wang H Hu J Hu H. Lateral habenula in the pathophysiology of depression. *Curr Opin Neurobiol.* (2018) 48:90–6. 10.1016/j.conb.2017.10.024 29175713

[10] Sutherland RJ. The dorsal diencephalic conduction system: a review of the anatomy and functions of the habenular complex. *Neurosci Biobehav R.* (1982) 6:1. 10.1016/0149-7634(82)90003-3 7041014

[11] Wang RY Aghajanian GK. Physiological evidence for habenula as major link between forebrain and midbrain raphe. *Science.* (1977) 197:89–91. 10.1126/science.194312 194312

[12] Christoph GR Leonzio RJ Wilcox KS. Stimulation of the lateral habenula inhibits dopamine-containing neurons in the substantia nigra and ventral tegmental area of the rat. *J Neurosci.* (1986) 6:613–9. 10.1523/JNEUROSCI.06-03-00613.1986 3958786 PMC6568453

[13] Li Y Wang Y Xuan C Li Y Piao L Li J Role of the lateral habenula in pain-associated depression. *Front Behav Neurosci.* (2017) 11:31. 10.3389/fnbeh.2017.00031 28270756 PMC5318408

[14] Li J Li Y Zhang B Shen X Zhao H. Why depression and pain often coexist and mutually reinforce: role of the lateral habenula. *Exp Neurol.* (2016) 284:106–13. 10.1016/j.expneurol.2016.08.010 27554829

[15] Stephenson-Jones M Yu K Ahrens S Tucciarone JM van Huijstee AN Mejia LA A basal ganglia circuit for evaluating action outcomes. *Nature.* (2016) 539:289–93. 10.1038/nature19845 27652894 PMC5161609

[16] Lammel S Lim BK Ran C Huang KW Betley MJ Tye KM Input-specific control of reward and aversion in the ventral tegmental area. *Nature.* (2012) 491:212–7. 10.1038/nature11527 23064228 PMC3493743

[17] Li K Zhou T Liao L Yang Z Wong C Henn F Bcamkii in lateral habenula mediates core symptoms of depression. *Science.* (2013) 341:1016–20. 10.1126/science.1240729 23990563 PMC3932364

[18] Huang L Xi Y Peng Y Yang Y Huang X Fu Y A visual circuit related to habenula underlies the antidepressive effects of light therapy. *Neuron.* (2019) 102:128–42. 10.1016/j.neuron.2019.01.037 30795900

[19] Cui Y Yang Y Ni Z Dong Y Cai G Foncelle A Astroglial kir4.1 in the lateral habenula drives neuronal bursts in depression. *Nature.* (2018) 554:323–7. 10.1038/nature25752 29446379

[20] Browne CA Hammack R Lucki I. Dysregulation of the lateral habenula in major depressive disorder. *Front Synaptic Neurosci.* (2018) 10:46. 10.3389/fnsyn.2018.00046 30581384 PMC6292991

[21] Gold PW Kadriu B. A major role for the lateral habenula in depressive illness: physiologic and molecular mechanisms. *Front Psychiatry.* (2019) 10:320. 10.3389/fpsyt.2019.00320 31231247 PMC6558383

[22] Concha ML Wilson SW. Asymmetry in the epithalamus of vertebrates. *J Anat.* (2001) 199:63–84. 10.1046/j.1469-7580.2001.19910063.x 11523830 PMC1594988

[23] Hétu S Luo Y Saez I D’Ardenne K Lohrenz T Montague PR. Asymmetry in functional connectivity of the human habenula revealed by high-resolution cardiac-gated resting state imaging. *Hum Brain Mapp.* (2016) 37:2602–15. 10.1002/hbm.23194 27038008 PMC4905773

[24] Ahumada-Galleguillos P Lemus CG Díaz E Osorio-Reich M Härtel S Concha ML. Directional asymmetry in the volume of the human habenula. *Brain Struct Funct.* (2017) 222:1087–92. 10.1007/s00429-016-1231-z 27155991

[25] Cho S Park C Na K Chung C Ma H Kang C Left-right asymmetric and smaller right habenula volume in major depressive disorder on high-resolution 7-t magnetic resonance imaging. *PLoS One.* (2021) 16:e255459. 10.1371/journal.pone.0255459 34343199 PMC8330903

[26] Cho S Kim N Na K Kang C Kang S. Thalamo-habenular connection differences between patients with major depressive disorder and normal controls. *Front Psychiatry.* (2021) 12:699416. 10.3389/fpsyt.2021.699416 34539461 PMC8440934

[27] Zhu Y Qi S Zhang B He D Teng Y Hu J Connectome-based biomarkers predict subclinical depression and identify abnormal brain connections with the lateral habenula and thalamus. *Front Psychiatry.* (2019) 10:371. 10.3389/fpsyt.2019.00371 31244688 PMC6581735

[28] Yang L Jin C Qi S Teng Y Li C Yao Y Alterations of functional connectivity of the lateral habenula in subclinical depression and major depressive disorder. *BMC Psychiatry.* (2022) 22:588. 10.1186/s12888-022-04221-6 36064380 PMC9442927

[29] Ichijo H Hamada M Takahashi S Kobayashi M Nagai T Toyama T Lateralization, maturation, and anteroposterior topography in the lateral habenula revealed by zif268/egr1 immunoreactivity and labeling history of neuronal activity. *Neurosci Res.* (2015) 95:27–37. 10.1016/j.neures.2015.01.005 25637311

[30] Liu W Valton V Wang L Zhu Y Roiser JP. Association between habenula dysfunction and motivational symptoms in unmedicated major depressive disorder. *Soc Cogn Affect Neur.* (2017) 12:1520–33. 10.1093/scan/nsx074 28575424 PMC5629818

[31] Cui W Zhang W Chen T Li Q Xu F Mao-Ying Q Tacr3 in the lateral habenula differentially regulates orofacial allodynia and anxiety-like behaviors in a mouse model of trigeminal neuralgia. *Acta Neuropathol Commun.* (2020) 8:44. 10.1186/s40478-020-00922-9 32264959 PMC7137530

[32] Moscicki MK Hurd PL. Damage-induced alarm cues influence lateralized behaviour but not the relationship between behavioural and habenular asymmetry in convict cichlids (*Amatitlania nigrofasciata*). *Anim Cogn.* (2017) 20:537–51. 10.1007/s10071-017-1081-y 28324234

[33] Hikosaka O. The habenula: from stress evasion to value-based decision-making. *Nat Rev Neurosci.* (2010) 11:503–13. 10.1038/nrn2866 20559337 PMC3447364

[34] Hikosaka O Sesack SR Lecourtier L Shepard PD. Habenula: crossroad between the basal ganglia and the limbic system. *J Neurosci.* (2008) 28:11825–9. 10.1523/JNEUROSCI.3463-08.2008 19005047 PMC2613689

[35] Gifuni AJ Jozaghi S Gauthier-Lamer AC Boye SM. Lesions of the lateral habenula dissociate the reward-enhancing and locomotor-stimulant effects of amphetamine. *Neuropharmacology.* (2012) 63:945–57. 10.1016/j.neuropharm.2012.07.032 22842070

[36] Zahm DS Root DH. Review of the cytology and connections of the lateral habenula, an avatar of adaptive behaving. *Pharmacol Biochem Behav.* (2017) 162:3–21. 10.1016/j.pbb.2017.06.004 28647565 PMC5659881

[37] Barker DJ Miranda-Barrientos J Zhang S Root DH Wang H Liu B Lateral preoptic control of the lateral habenula through convergent glutamate and GABA transmission. *Cell Rep.* (2017) 21:1757–69. 10.1016/j.celrep.2017.10.066 29141211 PMC5699228

[38] Webster JF Vroman R Balueva K Wulff P Sakata S Wozny C. Disentangling neuronal inhibition and inhibitory pathways in the lateral habenula. *Sci Rep.* (2020) 10:8490. 10.1038/s41598-020-65349-7 32444785 PMC7244525

[39] Liu B Cao Y Wang J Dong J. Excitatory transmission from ventral pallidum to lateral habenula mediates depression. *World J Biol Psychiatry.* (2020) 21:627–33. 10.1080/15622975.2020.1725117 32009492

[40] Golden SA Heshmati M Flanigan M Christoffel DJ Guise K Pfau ML Basal forebrain projections to the lateral habenula modulate aggression reward. *Nature.* (2016) 534:688–92. 10.1038/nature18601 27357796 PMC4930107

[41] Cui Y Huang X Huang P Huang L Feng Z Xiang X Reward ameliorates depressive-like behaviors via inhibition of the substantia innominata to the lateral habenula projection. *Sci Adv.* (2022) 8:n193. 10.1126/sciadv.abn0193 35857453 PMC9269896

[42] Shen L Zhang G Tao C Seo MB Zhang NK Huang JJ A bottom-up reward pathway mediated by somatostatin neurons in the medial septum complex underlying appetitive learning. *Nat Commun.* (2022) 13:1194. 10.1038/s41467-022-28854-z 35256596 PMC8901785

[43] Zhang G Shen L Zhong W Xiong Y Zhang LI Tao HW. Transforming sensory cues into aversive emotion via septal-habenular pathway. *Neuron.* (2018) 99:1016–28. 10.1016/j.neuron.2018.07.023 30122379 PMC6126968

[44] Liu X Huang H Zhang Y Wang L Wang F. Sexual dimorphism of inputs to the lateral habenula in mice. *Neurosci Bull.* (2022) [Epub ahead of print]. 10.1007/s12264-022-00885-y 35644002 PMC9723051

[45] Neugebauer V Mazzitelli M Cragg B Ji G Navratilova E Porreca F. Amygdala, neuropeptides, and chronic pain-related affective behaviors. *Neuropharmacology.* (2020) 170:108052. 10.1016/j.neuropharm.2020.108052 32188569 PMC7214122

[46] Shabel SJ Proulx CD Piriz J Malinow R. Mood regulation. Gaba/glutamate co-release controls habenula output and is modified by antidepressant treatment. *Science.* (2014) 345:1494–8. 10.1126/science.1250469 25237099 PMC4305433

[47] Lin S Huang L Luo ZC Li X Jin SY Du ZJ The ATP level in the medial prefrontal cortex regulates depressive-like behavior via the medial prefrontal cortex-lateral habenula pathway. *Biol Psychiatry.* (2022) 92:179–92. 10.1016/j.biopsych.2022.02.014 35489874

[48] Xu J Jo A DeVries RP Deniz S Cherian S Sunmola I Intersectional mapping of multi-transmitter neurons and other cell types in the brain. *Cell Rep.* (2022) 40:111036. 10.1016/j.celrep.2022.111036 35793636 PMC9290751

[49] Herkenham M Nauta WJH. Efferent connections of the habenular nuclei in the rat. *J Comp Neurol.* (1979) 187:19–47. 10.1002/cne.901870103 226566

[50] Metzger M Souza R Lima LB Bueno D Gonçalves L Sego C Habenular connections with the dopaminergic and serotonergic system and their role in stress-related psychiatric disorders. *Eur J Neurosci.* (2021) 53:65–88. 10.1111/ejn.14647 31833616

[51] Sun Y Cao J Xu C Liu X Wang Z Zhao H. Rostromedial tegmental nucleus-substantia nigra pars compacta circuit mediates aversive and despair behavior in mice. *Exp Neurol.* (2020) 333:113433. 10.1016/j.expneurol.2020.113433 32791155

[52] Quina LA Tempest L Ng L Harris JA Ferguson S Jhou TC Efferent pathways of the mouse lateral habenula. *J Comp Neurol.* (2015) 523:32–60. 10.1002/cne.23662 25099741 PMC4232452

[53] Purvis EM Klein AK Ettenberg A. Lateral habenular norepinephrine contributes to states of arousal and anxiety in male rats. *Behav Brain Res.* (2018) 347:108–15. 10.1016/j.bbr.2018.03.012 29526789 PMC5988948

[54] Yang H Yang J Xi W Hao S Luo B He X Laterodorsal tegmentum interneuron subtypes oppositely regulate olfactory cue-induced innate fear. *Nat Neurosci.* (2016) 19:283–9. 10.1038/nn.4208 26727549

[55] Li BO Piriz J Mirrione M Chung C Proulx CD Schulz D Synaptic potentiation onto habenula neurons in the learned helplessness model of depression. *Nature.* (2011) 470:535–9. 10.1038/nature09742 21350486 PMC3285101

[56] Szonyi A Zicho K Barth AM Gonczi RT Schlingloff D Torok B Median raphe controls acquisition of negative experience in the mouse. *Science.* (2019) 366:eaay8746. 10.1126/science.aay8746 31780530

[57] Xu Z Feng Z Zhao M Sun Q Deng L Jia X Whole-brain connectivity atlas of glutamatergic and gabaergic neurons in the mouse dorsal and median raphe nuclei. *Elife.* (2021) 10:e65502. 10.7554/eLife.65502 34792021 PMC8626088

[58] Zhang H Li K Chen H Gao S Xia Z Zhang J Dorsal raphe projection inhibits the excitatory inputs on lateral habenula and alleviates depressive behaviors in rats. *Brain Struct Funct.* (2018) 223:2243–58. 10.1007/s00429-018-1623-3 29460052

[59] Guan YF Huang GB Xu MD Gao F Lin S Huang J Anti-depression effects of ketogenic diet are mediated via the restoration of microglial activation and neuronal excitability in the lateral habenula. *Brain Behav Immun.* (2020) 88:748–62. 10.1016/j.bbi.2020.05.032 32413556

[60] Seo J Zhong P Liu A Yan Z Greengard P. Elevation of p11 in lateral habenula mediates depression-like behavior. *Mol Psychiatry.* (2018) 23:1113–9. 10.1038/mp.2017.96 28507317 PMC5690885

[61] Tchenio A Lecca S Valentinova K Mameli M. Limiting habenular hyperactivity ameliorates maternal separation-driven depressive-like symptoms. *Nat Commun.* (2017) 8:1135–8. 10.1038/s41467-017-01192-1 29074844 PMC5658350

[62] Fu Y Peng Y Huang X Yang Y Huang L Xi Y Lycium barbarum polysaccharide-glycoprotein preventative treatment ameliorates aversive. *Neural Regen Res.* (2021) 16:543. 10.4103/1673-5374.293156 32985485 PMC7996006

[63] Winter C Vollmayr B Djodari-Irani A Klein J Sartorius A. Pharmacological inhibition of the lateral habenula improves depressive-like behavior in an animal model of treatment resistant depression. *Behav Brain Res.* (2011) 216:463–5. 10.1016/j.bbr.2010.07.034 20678526

[64] Li Z Wang Y Zou H Jing X Liu Y Li L. Gaba(b) receptors within the lateral habenula modulate stress resilience and vulnerability in mice. *Physiol Behav.* (2021) 230:113311. 10.1016/j.physbeh.2021.113311 33412189

[65] Kim W Chung C. Brain-wide cellular mapping of acute stress-induced activation in male and female mice. *FASEB J.* (2021) 35:e22041. 10.1096/fj.202101287R 34780680

[66] Park H Rhee J Park K Han JS Malinow R Chung C. Exposure to stressors facilitates long-term synaptic potentiation in the lateral habenula. *J Neurosci.* (2017) 37:6021–30. 10.1523/JNEUROSCI.2281-16.2017 28539417 PMC6596499

[67] Congiu M Trusel M Pistis M Mameli M Lecca S. Opposite responses to aversive stimuli in lateral habenula neurons. *Eur J Neurosci.* (2019) 50:2921–30. 10.1111/ejn.14400 30860301

[68] Okamura H Yasugaki S Suzuki-Abe H Arai Y Sakurai K Yanagisawa M Long-term effects of repeated social defeat stress on brain activity during social interaction in balb/c mice. *eNeuro.* (2022) 9:0068-22. 10.1523/ENEURO.0068-22.2022 35437264 PMC9070729

[69] Stanisavljeviæ A Periæ I Gass P Inta D Lang UE Borgwardt S Fluoxetine modulates neuronal activity in stress-related limbic areas of adult rats subjected to the chronic social isolation. *Brain Res Bull.* (2020) 163:95–108. 10.1016/j.brainresbull.2020.07.021 32730865

[70] Karanges EA Ramos L Dampney B Suraev AS Li KM McGregor IS Contrasting regional FOS expression in adolescent and young adult rats following acute administration of the antidepressant paroxetine. *Brain Res Bull.* (2016) 121:246–54. 10.1016/j.brainresbull.2016.02.008 26876759

[71] Li J Chen P Han X Zuo W Mei Q Bian EY Differences between male and female rats in alcohol drinking, negative affects and neuronal activity after acute and prolonged abstinence. *Int J Physiol Pathophysiol Pharmacol.* (2019) 11:163–76. 31523363 PMC6737432

[72] Zhang S Zhang H Ku SM Juarez B Morel C Tzavaras N Sex differences in the neuroadaptations of reward-related circuits in response to subchronic variable stress. *Neuroscience.* (2018) 376:108–16. 10.1016/j.neuroscience.2018.02.021 29476894 PMC5860998

[73] Riecher-Rössler A. Sex and gender differences in mental disorders. *Lancet Psychiatry.* (2016) 4:8–9. 10.1016/S2215-0366(16)30348-0 27856397

[74] Sood A Chaudhari K Vaidya VA. Acute stress evokes sexually dimorphic, stressor-specific patterns of neural activation across multiple limbic brain regions in adult rats. *Stress.* (2018) 21:136–50. 10.1080/10253890.2017.1422488 29316846

[75] Langlois LD Berman RY Shepard RD Simmons SC Tsuda MC Gouty S Potentiation of glutamatergic synaptic transmission onto lateral habenula neurons following early life stress and intravenous morphine self-administration in rats. *Addict Biol.* (2021) 27:e13064. 10.1111/adb.13064 34036710 PMC8613295

[76] Cui Y Hu S Hu H. Lateral habenular burst firing as a target of the rapid antidepressant effects of ketamine. *Trends Neurosci.* (2019) 42:179–91. 10.1016/j.tins.2018.12.002 30823984

[77] Yang Y Cui Y Sang K Dong Y Ni Z Ma S Ketamine blocks bursting in the lateral habenula to rapidly relieve depression. *Nature.* (2018) 554:317–22. 10.1038/nature25509 29446381

[78] Shepard RD Langlois LD Browne CA Berenji A Lucki I Nugent FS. Ketamine reverses lateral habenula neuronal dysfunction and behavioral immobility in the forced swim test following maternal deprivation in late adolescent rats. *Front Synaptic Neurosci.* (2018) 10:39. 10.3389/fnsyn.2018.00039 30425634 PMC6218426

[79] Cerniauskas I Winterer J de Jong JW Lukacsovich D Yang H Khan F Chronic stress induces activity, synaptic, and transcriptional remodeling of the lateral habenula associated with deficits in motivated behaviors. *Neuron.* (2019) 104:899–915. 10.1016/j.neuron.2019.09.005 31672263 PMC6895430

[80] Durieux L Herbeaux K Borcuk C Cecile H Andry V Goumon Y Functional brain-wide network mapping during acute stress exposure in rats: interaction between the lateral habenula and cortical, amygdalar, hypothalamic and monoaminergic regions. *bioRxiv.* (2022) [Preprint]. 10.1101/2022.05.10.491280 35993349

[81] Clemm Von Hohenberg C Weber-Fahr W Lebhardt P Ravi N Braun U Gass N Lateral habenula perturbation reduces default-mode network connectivity in a rat model of depression. *Transl Psychiatry.* (2018) 8:68. 10.1038/s41398-018-0121-y 29581421 PMC5913319

[82] Parekh PK Johnson SB Liston C. Synaptic mechanisms regulating mood state transitions in depression. *Annu Rev Neurosci.* (2022) 45:581–601. 10.1146/annurev-neuro-110920-040422 35508195 PMC11577286

[83] Knowland D Lilascharoen V Pacia CP Shin S Wang EH Lim BK. Distinct ventral pallidal neural populations mediate separate symptoms of depression. *Cell.* (2017) 170:284–97. 10.1016/j.cell.2017.06.015 28689640 PMC5621481

[84] Pribiag H Shin S Wang EH Sun F Datta P Okamoto A Ventral pallidum drd3 potentiates a pallido-habenular circuit driving accumbal dopamine release and cocaine seeking. *Neuron.* (2021) 109:2165–82. 10.1016/j.neuron.2021.05.002 34048697 PMC9013317

[85] Cui Y Lv G Jin S Peng J Yuan J He X A central amygdala-substantia innominata neural circuitry encodes aversive reinforcement signals. *Cell Rep.* (2017) 21:1770–82. 10.1016/j.celrep.2017.10.062 29141212

[86] Francis TC Lobo MK. Emerging role for nucleus accumbens medium spiny neuron subtypes in depression. *Biol Psychiatry.* (2017) 81:645–53. 10.1016/j.biopsych.2016.09.007 27871668 PMC5352537

[87] Lecca S Meye FJ Trusel M Tchenio A Harris J Schwarz MK Aversive stimuli drive hypothalamus-to-habenula excitation to promote escape behavior. *Elife.* (2017) 6:e30697. 10.7554/eLife.30697 28871962 PMC5606847

[88] Lazaridis I Tzortzi O Weglage M Märtin A Xuan Y Parent M A hypothalamus-habenula circuit controls aversion. *Mol Psychiatry.* (2019) 24:1351–68. 10.1038/s41380-019-0369-5 30755721 PMC6756229

[89] Zheng Z Guo C Li M Yang L Liu P Zhang X Hypothalamus-habenula potentiation encodes chronic stress experience and drives depression onset. *Neuron.* (2022) 110:1400–15.e6. 10.1016/j.neuron.2022.01.011 35114101

[90] Warden MR Selimbeyoglu A Mirzabekov JJ Lo M Thompson KR Kim S A prefrontal cortex—brainstem neuronal projection that controls response to behavioural challenge. *Nature.* (2012) 492:428–32. 10.1038/nature11617 23160494 PMC5929119

[91] Benekareddy M Stachniak TJ Bruns A Knoflach F von Kienlin M Kunnecke B Identification of a corticohabenular circuit regulating socially directed behavior. *Biol Psychiatry.* (2018) 83:607–17. 10.1016/j.biopsych.2017.10.032 29336819

[92] Huang Y Sun B Debarros J Zhang C Zhan S Li D Increased theta/alpha synchrony in the habenula-prefrontal network with negative emotional stimuli in human patients. *Elife.* (2021) 10:e65444. 10.7554/eLife.65444 34251338 PMC8275130

[93] Zhang L Hernández VS Vázquez-Juárez E Chay FK Barrio RA. Thirst is associated with suppression of habenula output and active stress coping: is there a role for a non-canonical vasopressin-glutamate pathway? *Front Neural Circuit.* (2016) 10:13. 10.3389/fncir.2016.00013 27065810 PMC4814529

[94] Hastings MH Maywood ES Brancaccio M. Generation of circadian rhythms in the suprachiasmatic nucleus. *Nat Rev Neurosci.* (2018) 19:453–69. 10.1038/s41583-018-0026-z 29934559

[95] Young CJ Lyons D Piggins HD. Circadian influences on the habenula and their potential contribution to neuropsychiatric disorders. *Front Behav Neurosci.* (2022) 15:815700. 10.3389/fnbeh.2021.815700 35153695 PMC8831701

[96] Li Y Li G Li J Cai X Sun Y Zhang B Depression-like behavior is associated with lowerPer2 mRNA expression in the lateral habenula of rats. *Genes Brain Behav.* (2021) 20:e12702. 10.1111/gbb.12702 32964673

[97] Olejniczak I Ripperger JA Sandrelli F Schnell A Mansencal-Strittmatter L Wendrich K Light affects behavioral despair involving the clock gene period 1. *PLoS Genet.* (2021) 17:e1009625. 10.1371/journal.pgen.1009625 34237069 PMC8266116

[98] Sparta DR Jennings JH Ung RL Stuber GD. Optogenetic strategies to investigate neural circuitry engaged by stress. *Behav Brain Res.* (2013) 255:19–25. 10.1016/j.bbr.2013.05.007 23684554 PMC4415682

[99] Clauss JA Avery SN Benningfield MM Blackford JU. Social anxiety is associated with BNST response to unpredictability. *Depress Anxiety.* (2019) 36:666–75. 10.1002/da.22891 30953446 PMC6679811

[100] Bueno D Lima LB Souza R Goncalves L Leite F Souza S Connections of the laterodorsal tegmental nucleus with the habenular-interpeduncular-raphe system. *J Comp Neurol.* (2019) 527:3046–72. 10.1002/cne.24729 31199515

[101] Mao CP Chen FR Huo JH Zhang L Zhang GR Zhang B Altered resting-state functional connectivity and effective connectivity of the habenula in irritable bowel syndrome: a cross-sectional and machine learning study. *Hum Brain Mapp.* (2020) 41:3655–66. 10.1002/hbm.25038 32488929 PMC7416021

[102] Llorca-Torralba M Camarena-Delgado C Suarez-Pereira I Bravo L Mariscal P Garcia-Partida JA Pain and depression comorbidity causes asymmetric plasticity in the locus coeruleus neurons. *Brain.* (2022) 145:154–67. 10.1093/brain/awab239 34373893 PMC8967092

[103] Root DH Zhang S Barker DJ Miranda-Barrientos J Liu B Wang H Selective brain distribution and distinctive synaptic architecture of dual glutamatergic-gabaergic neurons. *Cell Rep.* (2018) 23:3465–79. 10.1016/j.celrep.2018.05.063 29924991 PMC7534802

[104] Kim S Wallace ML El-Rifai M Knudsen AR Sabatini BL. Co-packaging of opposing neurotransmitters in individual synaptic vesicles in the central nervous system. *Neuron.* (2022) 110:1371–84. 10.1016/j.neuron.2022.01.007 35120627 PMC9056948

[105] Meye FJ Soiza-Reilly M Smit T Diana MA Schwarz MK Mameli M. Shifted pallidal co-release of GABA and glutamate in habenula drives cocaine withdrawal and relapse. *Nat Neurosci.* (2016) 19:1019–24. 10.1038/nn.4334 27348214

[106] Yang Y Liu J Wang Y Wu X Li L Bian G Blockade of pre-synaptic and post-synaptic GABAB receptors in the lateral habenula produces different effects on anxiety-like behaviors in 6-hydroxydopamine hemiparkinsonian rats. *Neuropharmacology.* (2021) 196:108705. 10.1016/j.neuropharm.2021.108705 34246684

[107] Li J Fan R Liu X Shen X Liu X Zhao H. The convergence of aversion and reward signals in individual neurons of the mice lateral habenula. *Exp Neurol.* (2021) 339:113637. 10.1016/j.expneurol.2021.113637 33549547

[108] Stephenson-Jones M Bravo-Rivera C Ahrens S Furlan A Xiao X Fernandes-Henriques C Opposing contributions of gabaergic and glutamatergic ventral pallidal neurons to motivational behaviors. *Neuron.* (2020) 105:921–33. 10.1016/j.neuron.2019.12.006 31948733 PMC8573387

[109] Zhu C Yao Y Xiong Y Cheng M Chen J Zhao R Somatostatin neurons in the basal forebrain promote high-calorie food intake. *Cell Rep.* (2017) 20:112–23. 10.1016/j.celrep.2017.06.007 28683305

[110] Webster JF Lecca S Wozny C. Inhibition within the lateral habenula-implications for affective disorders. *Front Behav Neurosci.* (2021) 15:786011. 10.3389/fnbeh.2021.786011 34899206 PMC8661446

[111] Meye FJ Lecca S Valentinova K Mameli M. Synaptic and cellular profile of neurons in the lateral habenula. *Front Hum Neurosci.* (2013) 7:860. 10.3389/fnhum.2013.00860 24379770 PMC3863943

[112] Good CH Wang H Chen Y Mejias-Aponte CA Hoffman AF Lupica CR. Dopamine d4 receptor excitation of lateral habenula neurons via multiple cellular mechanisms. *J Neurosci.* (2013) 33:16853–64. 10.1523/JNEUROSCI.1844-13.2013 24155292 PMC3807019

[113] Matsumoto M Hikosaka O. Lateral habenula as a source of negative reward signals in dopamine neurons. *Nature.* (2007) 447:1111–5. 10.1038/nature05860 17522629

[114] Brown PL Palacorolla H Brady D Riegger K Elmer GI Shepard PD. Habenula-induced inhibition of midbrain dopamine neurons is diminished by lesions of the rostromedial tegmental nucleus. *J Neurosci.* (2017) 37:217–25. 10.1523/JNEUROSCI.1353-16.2016 28053043 PMC5214632

[115] Tye KM Mirzabekov JJ Warden MR Ferenczi EA Tsai H Finkelstein J Dopamine neurons modulate neural encoding and expression of depression-related behaviour. *Nature.* (2013) 493:537–41. 10.1038/nature11740 23235822 PMC4160519

[116] Moreines JL Owrutsky ZL Grace AA. Involvement of infralimbic prefrontal cortex but not lateral habenula in dopamine attenuation after chronic mild stress. *Neuropsychopharmacology.* (2017) 42:904–13. 10.1038/npp.2016.249 27813530 PMC5312072

[117] Brown PL Shepard PD. Functional evidence for a direct excitatory projection from the lateral habenula to the ventral tegmental area in the rat. *J Neurophysiol.* (2016) 116:1161–74. 10.1152/jn.00305.2016 27358317 PMC5013172

[118] Lyu S Guo Y Zhang L Tang G Li R Yang J Downregulation of astroglial glutamate transporter glt-1 in the lateral habenula is associated with depressive-like behaviors in a rat model of Parkinson’s disease. *Neuropharmacology.* (2021) 196:108691. 10.1016/j.neuropharm.2021.108691 34197892

[119] Lyu S Guo Y Zhang L Wang Y Tang G Li R Blockade of GABA transporter-1 and GABA transporter-3 in the lateral habenula improves depressive-like behaviors in a rat model of Parkinson’s disease. *Neuropharmacology.* (2020) 181:108369. 10.1016/j.neuropharm.2020.108369 33096108

[120] Metzger M Bueno D Lima LB. The lateral habenula and the serotonergic system. *Pharmacol Biochem Behav.* (2017) 162:22–8. 10.1016/j.pbb.2017.05.007 28528079

[121] Zhou L Liu MZ Li Q Deng J Mu D Sun YG. Organization of functional long-range circuits controlling the activity of serotonergic neurons in the dorsal raphe nucleus. *Cell Rep.* (2017) 18:3018–32. 10.1016/j.celrep.2017.02.077 28329692

[122] Sego C Goncalves L Lima L Furigo IC Donato JJ Metzger M. Lateral habenula and the rostromedial tegmental nucleus innervate neurochemically distinct subdivisions of the dorsal raphe nucleus in the rat. *J Comp Neurol.* (2014) 522:1454–84. 10.1002/cne.23533 24374795

[123] Andalman AS Burns VM Lovett-Barron M Broxton M Poole B Yang SJ Neuronal dynamics regulating brain and behavioral state transitions. *Cell.* (2019) 177:970–85. 10.1016/j.cell.2019.02.037 31031000 PMC6726130

[124] Liu H Rastogi A Narain P Xu Q Sabanovic M Alhammadi AD Blunted diurnal firing in lateral habenula projections to dorsal raphe nucleus and delayed photoentrainment in stress-susceptible mice. *PLoS Biol.* (2021) 19:e3000709. 10.1371/journal.pbio.3000709 33690628 PMC7984642

[125] Shen X Yuan H Wang G Xue H Liu Y Zhang C. Role of DNA hypomethylation in lateral habenular nucleus in the development of depressive-like behavior in rats. *J Affect Disorders.* (2019) 252:373–81. 10.1016/j.jad.2019.03.062 30999094

[126] Yang L Yu L Jin H Zhao H. Substance p receptor antagonist in lateral habenula improves rat depression-like behavior. *Brain Res Bull.* (2014) 100:22–8. 10.1016/j.brainresbull.2013.10.007 24157953

[127] Takahashi A Cuttoli RD Flanigan ME Hasegawa E Tsunematsu T Aleyasin H Lateral habenula glutamatergic neurons projecting to the dorsal raphe nucleus promote aggressive arousal in mice. *Nat Commun.* (2022) 13:1–18. 10.1038/s41467-022-31728-z 35864121 PMC9304121

[128] Zhou W Jin Y Meng Q Zhu X Bai T Tian Y A neural circuit for comorbid depressive symptoms in chronic pain. *Nat Neurosci.* (2019) 22:1649–58. 10.1038/s41593-019-0468-2 31451801

[129] Flanigan ME Aleyasin H Li L Burnett CJ Chan KL LeClair KB Orexin signaling in gabaergic lateral habenula neurons modulates aggressive behavior in male mice. *Nat Neurosci.* (2020) 23:638–50. 10.1038/s41593-020-0617-7 32284606 PMC7195257

[130] Peyron C Tighe DK van den Pol AN de Lecea L Heller HC Sutcliffe JG Neurons containing hypocretin (orexin) project to multiple neuronal systems. *J Neurosci.* (1998) 18:9996–10015. 9822755 10.1523/JNEUROSCI.18-23-09996.1998 PMC6793310

[131] Wang D Li A Dong K Li H Guo Y Zhang X Lateral hypothalamus orexinergic inputs to lateral habenula modulate maladaptation after social defeat stress. *Neurobiol Stress.* (2021) 14:100298. 10.1016/j.ynstr.2021.100298 33569507 PMC7859368

[132] Aizawa H Kobayashi M Tanaka S Fukai T Okamoto H. Molecular characterization of the subnuclei in rat habenula. *J Comp Neurol.* (2012) 520:4051–66. 10.1002/cne.23167 22700183

[133] Henter ID de Sousa RT Zarate JCA. Glutamatergic modulators in depression. *Harvard Rev Psychiatry.* (2018) 26:307–19. 10.1097/HRP.0000000000000183 29465478 PMC6102095

[134] Kadriu B Musazzi L Henter ID Graves M Popoli M Zarate JCA. Glutamatergic neurotransmission: pathway to developing novel rapid-acting antidepressant treatments. *Int J Neuropsychopharmacol.* (2019) 22:119–35. 10.1093/ijnp/pyy094 30445512 PMC6368372

[135] Tian Y Wu Z Wang Y Chen C He Y Lan T Alterations of neurotransmitters and related metabolites in the habenula from CUMS-susceptible and -resilient rats. *Biochem Biophys Res Commun.* (2021) 534:422–8. 10.1016/j.bbrc.2020.11.065 33246560

[136] Kang S Li J Bekker A Ye JH. Rescue of glutamate transport in the lateral habenula alleviates depression- and anxiety-like behaviors in ethanol-withdrawn rats. *Neuropharmacology.* (2018) 129:47–56. 10.1016/j.neuropharm.2017.11.013 29128307 PMC5714683

[137] Nuno-Perez A Trusel M Lalive AL Congiu M Gastaldo D Tchenio A Stress undermines reward-guided cognitive performance through synaptic depression in the lateral habenula. *Neuron.* (2021) 109:947–56. 10.1016/j.neuron.2021.01.008 33535028 PMC7980092

[138] Shi B Luo J Fang Y Liu X Rao Z Liu R Xiaoyao pills prevent lipopolysaccharide-induced depression by inhibiting inflammation and protecting nerves. *Front Pharmacol.* (2019) 10:1324. 10.3389/fphar.2019.01324 31798446 PMC6863983

[139] Gakare SG Varghese SS Patni PP Wagh SA Ugale RR. Prevention of glutamate excitotoxicity in lateral habenula alleviates ethanol withdrawal-induced somatic and behavioral effects in ethanol dependent mice. *Behav Brain Res.* (2022) 416:113557. 10.1016/j.bbr.2021.113557 34453973

[140] Lei T Dong D Song M Sun Y Liu X Zhao H. Rislenemdaz treatment in the lateral habenula improves despair-like behavior in mice. *Neuropsychopharmacology.* (2020) 45:1717–24. 10.1038/s41386-020-0652-9 32147667 PMC7419533

[141] Kang M Noh J Chung J. Nmda receptor-dependent long-term depression in the lateral habenula: implications in physiology and depression. *Sci Rep.* (2020) 10:17921. 10.1038/s41598-020-74496-w 33087756 PMC7578045

[142] Bredt DS Nicoll RA. AMPA receptor trafficking at excitatory synapses. *Neuron.* (2003) 40:361–79. 10.1016/S0896-6273(03)00640-8 14556714

[143] Zhang J Lv S Tang G Bian G Yang Y Li R Activation of calcium-impermeable glur2-containing AMPA receptors in the lateral habenula produces antidepressant-like effects in a rodent model of Parkinson’s disease. *Exp Neurol.* (2019) 322:113058. 10.1016/j.expneurol.2019.113058 31499061

[144] Zhang J Wang Y Sun YN Li LB Zhang L Guo Y Blockade of calcium-permeable AMPA receptors in the lateral habenula produces increased antidepressant-like effects in unilateral 6-hydroxydopamine-lesioned rats compared to sham-lesioned rats. *Neuropharmacology.* (2019) 157:107687. 10.1016/j.neuropharm.2019.107687 31251995

[145] Shor C Zuo W Eloy JD Ye J. The emerging role of LHb CaMKII in the comorbidity of depressive and alcohol use disorders. *Int J Mol Sci.* (2020) 21:8123. 10.3390/ijms21218123 33143210 PMC7663385

[146] Quina LA Walker A Morton G Han V Turner EE. Gad2 expression defines a class of excitatory lateral habenula neurons in mice that project to the raphe and pontine tegmentum. *eNeuro.* (2020) 7:0527-19. 10.1523/ENEURO.0527-19.2020 32332079 PMC7240287

[147] Zhang L Hernández VS Swinny JD Verma AK Giesecke T Emery AC A gabaergic cell type in the lateral habenula links hypothalamic homeostatic and midbrain motivation circuits with sex steroid signaling. *Transl Psychiatry.* (2018) 8:50. 10.1038/s41398-018-0099-5 29479060 PMC5865187

[148] Ramaswamy M Cheng RK Jesuthasan S. Identification of gabaergic neurons innervating the zebrafish lateral habenula. *Eur J Neurosci.* (2020) 52:3918–28. 10.1111/ejn.14843 32464693 PMC7689879

[149] Nakamura T Kurosaki K Kanemoto M Sasahara M Ichijo H. Early-life experiences altered the maturation of the lateral habenula in mouse models, resulting in behavioural disorders in adulthood. *J Psychiatry Neurosci.* (2021) 46:E480–9. 10.1503/jpn.200226 34346201 PMC8410472

[150] Siemian JN Arenivar MA Sarsfield S Borja CB Erbaugh LJ Eagle AL An excitatory lateral hypothalamic circuit orchestrating pain behaviors in mice. *Elife.* (2021) 10:e66446. 10.7554/eLife.66446 34042586 PMC8159376

[151] Wang T Zhang L Zhang Q Wang Y Du C Sun Y Involvement of lateral habenula α1 subunit-containing GABA _*A*_ receptor-mediated inhibitory transmission in the regulation of depression-related behaviors in experimental Parkinson’s disease. *Neuropharmacology.* (2017) 116:399–411. 10.1016/j.neuropharm.2017.01.015 28109827

[152] Lalive AL Congiu M Lewis C Groos D Clerke JA Tchenio A Synaptic inhibition in the lateral habenula shapes reward anticipation. *Curr Biol.* (2022) 32:1829–36. 10.1016/j.cub.2022.02.035 35259343

[153] Lecca S Pelosi A Tchenio A Moutkine I Lujan R Hervé D Rescue of GABAB and GIRK function in the lateral habenula by protein phosphatase 2A inhibition ameliorates depression-like phenotypes in mice. *Nat Med.* (2016) 22:254–61. 10.1038/nm.4037 26808347

[154] Lalive AL Nuno Perez A Tchenio A Mameli M. Mild stress accumulation limits gabaergic synaptic plasticity in the lateral habenula. *Eur J Neurosci.* (2022) 55:377–87. 10.1111/ejn.15581 34963191 PMC9305738

[155] Lee YA Kim YJ Lee JS Lee S Goto Y. Imbalance between dopamine and serotonin caused by neonatal habenula lesion. *Behav Brain Res.* (2021) 409:113316. 10.1016/j.bbr.2021.113316 33901435

[156] Kowski AB Veh RW Weiss T. Dopaminergic activation excites rat lateral habenular neurons in vivo. *Neuroscience.* (2009) 161:1154–65. 10.1016/j.neuroscience.2009.04.026 19374940

[157] Chan J Ni Y Zhang P Chen Y Zhang J. D1-like dopamine receptor dysfunction in the lateral habenula nucleus increased anxiety-like behavior in rat. *Neuroscience.* (2016) 340:542–50. 10.1016/j.neuroscience.2016.11.005 27865867

[158] Hui Y Du C Xu T Zhang Q Tan H Liu J. Dopamine D_4_ receptors in the lateral habenula regulate depression-related behaviors via a pre-synaptic mechanism in experimental Parkinson’s disease. *Neurochem Int.* (2020) 140:104844. 10.1016/j.neuint.2020.104844 32891683

[159] Ochi T Vyalova NM Losenkov IS Paderina DZ Pozhidaev IV Loonen A Preliminary pharmacogenetic study to explore putative dopaminergic mechanisms of antidepressant action. *J Pers Med.* (2021) 11:731. 10.3390/jpm11080731 34442374 PMC8401614

[160] Okaty BW Commons KG Dymecki SM. Embracing diversity in the 5-ht neuronal system. *Nat Rev Neurosci.* (2019) 20:397–424. 10.1038/s41583-019-0151-3 30948838

[161] Delicata F Bombardi C Pierucci M Di Maio R De Deurwaerdère P Di Giovanni G. Preferential modulation of the lateral habenula activity by serotonin-2A rather than -2C receptors: electrophysiological and neuroanatomical evidence. *CNS Neurosci Ther.* (2018) 24:721–33. 10.1111/cns.12830 29479825 PMC6490100

[162] Hwang E Chung J. 5HT1B receptor-mediated pre-synaptic depression of excitatory inputs to the rat lateral habenula. *Neuropharmacology.* (2014) 81:153–65. 10.1016/j.neuropharm.2014.01.046 24508708

[163] Guo Y Zhang L Zhang J Lv S Du C Wang T Activation and blockade of serotonin-4 receptors in the lateral habenula produce antidepressant effects in the hemiparkinsonian rat. *Neuropsychobiology.* (2021) 80:52–63. 10.1159/000508680 32663830

[164] Han LN Zhang L Sun YN Du CX Zhang YM Wang T Serotonin7 receptors in the lateral habenular nucleus regulate depressive-like behaviors in the hemiparkinsonian rats. *Brain Res.* (2016) 1644:79–87. 10.1016/j.brainres.2016.05.016 27178363

[165] Han L Zhang L Li L Sun Y Wang Y Chen L Activation of serotonin(2c) receptors in the lateral habenular nucleus increases the expression of depression-related behaviors in the hemiparkinsonian rat. *Neuropharmacology.* (2015) 93:68–79. 10.1016/j.neuropharm.2015.01.024 25661701

[166] Mansour A Fox CA Akil H Watson SJ. Opioid-receptor mRNA expression in the rat CNS: anatomical and functional implications. *Trends Neurosci.* (1995) 18:22–9. 10.1016/0166-2236(95)93946-u 7535487

[167] Clarke S Chen Z Hsu M Pintar J Hill R Kitchen I. Quantitative autoradiographic mapping of the orl1, μ-, δ- and κ-receptors in the brains of knockout mice lacking the orl1 receptor gene. *Brain Res.* (2001) 906:13–24. 10.1016/S0006-8993(01)02531-8 11430857

[168] Margolis EB Fields HL. Mu opioid receptor actions in the lateral habenula. *PLoS One.* (2016) 11:e159097. 10.1371/journal.pone.0159097 27427945 PMC4948872

[169] Waung MW Maanum KA Cirino TJ Driscoll JR O’Brien C Bryant S A diencephalic circuit in rats for opioid analgesia but not positive reinforcement. *Nat Commun.* (2022) 13:764. 10.1038/s41467-022-28332-6 35140231 PMC8828762

[170] Chen C Willhouse AH Huang P Ko N Wang Y Xu B Characterization of a knock-in mouse line expressing a fusion protein of κ opioid receptor conjugated with tdtomato: 3-dimensional brain imaging via clarity. *eNeuro.* (2020) 7:0028-20. 10.1523/ENEURO.0028-20.2020 32561573 PMC7385665

[171] Simmons SC Shepard RD Gouty S Langlois LD Flerlage WJ Cox BM Early life stress dysregulates kappa opioid receptor signaling within the lateral habenula. *Neurobiol Stress.* (2020) 13:100267. 10.1016/j.ynstr.2020.100267 33344720 PMC7739170

[172] Welsch L Bailly J Darcq E Kieffer BL. The negative affect of protracted opioid abstinence: progress and perspectives from rodent models. *Biol Psychiatry.* (2020) 87:54–63. 10.1016/j.biopsych.2019.07.027 31521334 PMC6898775

[173] Klein ME Chandra J Sheriff S Malinow R. Opioid system is necessary but not sufficient for antidepressive actions of ketamine in rodents. *Proc Natl Acad Sci USA.* (2020) 117:2656–62. 10.1073/pnas.1916570117 31941713 PMC7007545

[174] Morales-Medina JC Dumont Y Quirion R. A possible role of neuropeptide y in depression and stress. *Brain Res.* (2009) 1314:194–205. 10.1016/j.brainres.2009.09.077 19782662

[175] Sundström G Larsson TA Xu B Heldin J Larhammar D. Interactions of zebrafish peptide YYb with the neuropeptide y-family receptors Y4, Y7, Y8a, and Y8b. *Front Neurosci Switz.* (2013) 7:29. 10.3389/fnins.2013.00029 23508731 PMC3598007

[176] Cheon M Park H Rhim H Chung C. Actions of neuropeptide y on synaptic transmission in the lateral habenula. *Neuroscience.* (2019) 410:183–90. 10.1016/j.neuroscience.2019.04.053 31082535

[177] Cheon M Park H Chung C. Protein kinase c mediates neuropeptide y-induced reduction in inhibitory neurotransmission in the lateral habenula. *Neuropharmacology.* (2020) 180:108295. 10.1016/j.neuropharm.2020.108295 32882226

[178] Rezitis J Herzog H Ip CK. Neuropeptide y interaction with dopaminergic and serotonergic pathways: interlinked neurocircuits modulating hedonic eating behaviours. *Prog Neuropsychopharmacol Biol Psychiatry.* (2022) 113:110449. 10.1016/j.pnpbp.2021.110449 34592387

[179] Jimenez AJ Mancera JM Perez-Figares JM Fernandez-Llebrez P. Distribution of galanin-like immunoreactivity in the brain of the turtle mauremys caspica. *J Comp Neurol.* (1994) 349:73–84. 10.1002/cne.903490106 7531723

[180] Alponti RF Mancera JM Martin-del-Rio MP Silveira PF. Galanin-like immunoreactivity in the brain of the snake bothrops jararaca. *Gen Comp Endocrinol.* (2006) 149:269–77. 10.1016/j.ygcen.2006.06.007 16860322

[181] García-Durán L Flores-Burgess A Cantero-García N Puigcerver A Narváez JÁ Fuxe K Galanin (1-15) potentiates the antidepressant-like effects induced by escitalopram in a rat model of depression. *Int J Mol Sci.* (2021) 22:10848. 10.3390/ijms221910848 34639188 PMC8509384

[182] Wagner F French L Veh RW. Transcriptomic-anatomic analysis of the mouse habenula uncovers a high molecular heterogeneity among neurons in the lateral complex, while gene expression in the medial complex largely obeys subnuclear boundaries. *Brain Struct Funct.* (2014) 221:39–58. 10.1007/s00429-014-0891-9 25244943

[183] Levinstein MR Bergkamp DJ Lewis ZK Tsobanoudis A Hashikawa K Stuber GD Pacap-expressing neurons in the lateral habenula diminish negative emotional valence. *Genes Brain Behav.* (2022) 21:e12801. 10.1111/gbb.12801 35304804 PMC9444940

[184] Hashikawa Y Hashikawa K Rossi MA Basiri ML Liu Y Johnston NL Transcriptional and spatial resolution of cell types in the mammalian habenula. *Neuron.* (2020) 106:743–58. 10.1016/j.neuron.2020.03.011 32272058 PMC7285796

[185] Lu Y Wang L Chen J Zhu J Meng X You Z Projections from lateral habenular to tail of ventral tegmental area contribute to inhibitory effect of stress on morphine-induced conditioned place preference. *Brain Res.* (2019) 1717:35–43. 10.1016/j.brainres.2019.03.026 30914248

[186] Brown JC Higgins ES George MS. Synaptic plasticity 101: the story of the ampa receptor for the brain stimulation practitioner. *Neuromodulation.* (2022) [Epub ahead of print]. 10.1016/j.neurom.2021.09.003 35088731 PMC10479373

[187] Li J Kang S Fu R Wu L Wu W Liu H Inhibition of AMPA receptor and CaMKII activity in the lateral habenula reduces depressive-like behavior and alcohol intake in rats. *Neuropharmacology.* (2017) 126:108–20. 10.1016/j.neuropharm.2017.08.035 28865912 PMC5634930

[188] Opazo P Labrecque S Tigaret CM Frouin A Wiseman PW De Koninck P CaMKII triggers the diffusional trapping of surface AMPARs through phosphorylation of stargazin. *Neuron.* (2010) 67:239–52. 10.1016/j.neuron.2010.06.007 20670832

[189] Zhang Q Feng JJ Yang S Liu XF Li JC Zhao H. Lateral habenula as a link between thyroid and serotoninergic system modiates depressive symptoms in hypothyroidism rats. *Brain Res Bull.* (2016) 124:198–205. 10.1016/j.brainresbull.2016.05.007 27185576

[190] Lisman J Yasuda R Raghavachari S. Mechanisms of CaMKII action in long-term potentiation. *Nat Rev Neurosci.* (2012) 13:169–82. 10.1038/nrn3192 22334212 PMC4050655

[191] Park H Rhee J Lee S Chung C. Selectively impaired endocannabinoid-dependent long-term depression in the lateral habenula in an animal model of depression. *Cell Rep.* (2017) 20:289–96. 10.1016/j.celrep.2017.06.049 28700932

[192] Mouro FM Ribeiro JA Sebastiao AM Dawson N. Chronic, intermittent treatment with a cannabinoid receptor agonist impairs recognition memory and brain network functional connectivity. *J Neurochem.* (2018) 147:71–83. 10.1111/jnc.14549 29989183 PMC6220860

[193] Authement ME Langlois LD Shepard RD Browne CA Lucki I Kassis H A role for corticotropin-releasing factor signaling in the lateral habenula and its modulation by early-life stress. *Sci Signal.* (2018) 11:eaan6480. 10.1126/scisignal.aan6480 29511121 PMC5861378

[194] Humburg BA Jordan CJ Zhang HY Shen H Han X Bi GH Optogenetic brain-stimulation reward: a new procedure to re-evaluate the rewarding versus aversive effects of cannabinoids in dopamine transporter-CRE mice. *Addict Biol.* (2021) 26:e13005. 10.1111/adb.13005 33538103 PMC9308103

[195] Arjmand S Landau AM Varastehmoradi B Andreatini R Joca S Wegener G. The intersection of astrocytes and the endocannabinoid system in the lateral habenula: on the fast-track to novel rapid-acting antidepressants. *Mol Psychiatry.* (2022) [Epube ahead of print]. 10.1038/s41380-022-01598-4 35585261

[196] Sartorius A Kiening KL Kirsch P von Gall CC Haberkorn U Unterberg AW Remission of major depression under deep brain stimulation of the lateral habenula in a therapy-refractory patient. *Biol Psychiatry.* (2010) 67:e9–11. 10.1016/j.biopsych.2009.08.027 19846068

[197] Meng H Wang Y Huang M Lin W Wang S Zhang B. Chronic deep brain stimulation of the lateral habenula nucleus in a rat model of depression. *Brain Res.* (2011) 1422:32–8. 10.1016/j.brainres.2011.08.041 21978548

[198] Zhou Q Dong J Xu T Cai X. Synaptic potentiation mediated by l-type voltage-dependent calcium channels mediates the antidepressive effects of lateral habenula stimulation. *Neuroscience.* (2017) 362:25–32. 10.1016/j.neuroscience.2017.08.025 28844005

[199] Kim Y Morath B Hu C Byrne LK Sutor SL Frye MA Antidepressant actions of lateral habenula deep brain stimulation differentially correlate with camkii/gsk3/ampk signaling locally and in the infralimbic cortex. *Behav Brain Res.* (2016) 306:170–7. 10.1016/j.bbr.2016.02.039 26956153

[200] He N Sethi SK Zhang C Li Y Chen Y Sun B Visualizing the lateral habenula using susceptibility weighted imaging and quantitative susceptibility mapping. *Magn Reson Imaging.* (2020) 65:55–61. 10.1016/j.mri.2019.09.005 31655137

